# Electrostatically Reinforced Double Network Granular Hydrogels

**DOI:** 10.1002/advs.202412566

**Published:** 2025-04-03

**Authors:** Tianyu Yuan, Chenzhuo Li, John M. Kolinski, Esther Amstad

**Affiliations:** ^1^ Soft Materials Laboratory Institute of Materials École Polytechnique Fédérale de Lausanne (EPFL) Lausanne 1015 Switzerland; ^2^ Engineering Mechanics of Soft Interfaces Laboratory Institute of Mechanical Engineering École Polytechnique Fédérale de Lausanne (EPFL) Lausanne 1015 Switzerland

**Keywords:** additive manufacturing, fracture energy, granular hydrogel, interfacial reinforcement

## Abstract

Rapid advances in biomedical applications and soft robotics demand load‐bearing soft materials that can be processed into complex 3D shapes. Direct ink writing (DIW) enables the fabrication of customizable shapes with locally varying compositions. Hydrogels that are formulated as microgels meet the rheological requirements that DIW imparts on the inks if they are jammed. However, most granular hydrogels are soft because inter‐particle interactions are weak. These hydrogels can be reinforced with a second hydrogel, yielding double network granular hydrogels (DNGHs). Yet, DNGHs suffer from low fracture energy. This limitation is addressed by electrostatically reinforcing them. The resulting materials exhibit Young's moduli and fracture energies similar to values of cartilage and muscles. An empirical model is proposed to predict the fracture energy of these reinforced DNGHs, based on the dissipation zone size, contact area, and adhesion energy. These DNGHs can be 3D‐N, N‐methylene bisacrylamideprinted into free‐standing structures exhibiting tuneable mechanical properties at the centimeter scale without the need for supporting structures.

## Graphical abstract

Electrostatically reinforced double network granular hydrogels (DNGHs) with cartilage‐like fracture energy are introduced. An empirical model predicts the composition‐dependent fracture energy based on dissipation zone size, contact area, and inter‐particle adhesion energy. Thanks to their granular structure and the inter‐particle attraction, the DNGHs can be processed into cm‐sized free‐standing structures via direct ink writing (DIW).

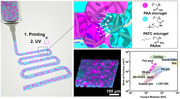

## 1 Introduction

Hydrogels are polymer networks that retain large amounts of water. They are widely used in the biomedical field and are increasingly often applied in soft robotics due to their responsiveness to external stimuli.^[^ ^1^, ^2^ ^]^ Unfortunately, conventional hydrogels suffer from low fracture energy, making them incompatible with load‐bearing applications. This shortcoming can be addressed through the introduction of effective energy dissipation mechanisms. Energy can be dissipated by fracturing bonds located within a certain volume around the crack tip, called process zone dissipation.^[^ ^3^ ^]^ This energy dissipation mechanism is present in hydrogels possessing homogeneous, isotropic chemical and physical properties.^[^ ^4^, ^5^ ^]^ In hydrogels, energy is most commonly dissipated by irreversibly breaking covalent bonds or reversibly dissociating physical bonds. Physical bonds are typically reversible yet, they are much weaker than covalent bonds, such that their dissociation energy is much lower. The energy dissipation within hydrogels has been increased by introducing sacrificial covalent bonds that break if sufficiently stretched.^[^ ^6^, ^7^ ^]^ For example, the toughness of double network (DN) hydrogels is enhanced because covalent bonds of the first network break within the dissipation zone before catastrophic crack propagation occurs.^[^ ^6^, ^8^ ^]^ However, the fabrication of double network hydrogels is limited to molding relatively simple structures.

Certain hydrogels can be processed into complex 3D structures possessing locally varying compositions through direct ink writing (DIW).^[^ ^9^ ^]^ However, DIW imposes strict rheological requirements on its inks, such as shear thinning properties, low yield points, and fast stress recovery.^[^ ^10^, ^11^ ^]^ These criteria limit the selection of hydrogel precursors suitable for direct ink writing. A much broader range of hydrogel formulations can be direct‐ink‐written if hydrogels are formulated as microgels.^[^ ^12^, ^13^ ^]^ Microgels inherently fulfill the rheological requirements of DIW if they are swollen and jammed.^[^ ^13^, ^14^ ^]^ However, the resulting granular hydrogels are typically rather soft due to weak inter‐particle interactions.^[^ ^15^ ^]^ The weak inter‐particle interactions can be strengthened through surface modification. Indeed, surface‐modified microgels have been connected via reversible bonds such as host–guest interactions,^[^ ^16^ ^]^ electrostatic interactions,^[^ ^17^, ^18^ ^]^ and hydrogen bonds.^[^ ^19^ ^]^ These reversible inter‐particle interactions can impart self‐healing properties to them.^[^ ^20^ ^]^ Yet, these reinforced granular hydrogels are still soft. Adjacent microgels have also been covalently connected through click chemistry or enzymatic catalysis.^[^ ^21^, ^22^ ^]^ However, even with these covalent connections, the granular hydrogels exhibited low mechanical strength, likely due to the limited contact area of adjacent microgels that results in a low density of inter‐particle links. Moreover, these hydrogels were rather brittle.

Granular hydrogels can be further toughened by covalently connecting microgels through a percolating hydrogel network that interpenetrates them, resulting in double network granular hydrogels (DNGHs).^[^ ^13^ ^]^ Within the microgels, DNGHs exhibit a double network structure whereas in the interstitial spaces DNGHs are composed of a single network.^[^ ^23^ ^]^ The dynamic fracture of bulk DNs has been systematically studied.^[^ ^6^, ^7^, ^24^ ^]^ By contrast, the fracture mechanisms of DNGHs remain elusive, making it challenging to intentionally adjust their fracture energy.

**Figure 1:**
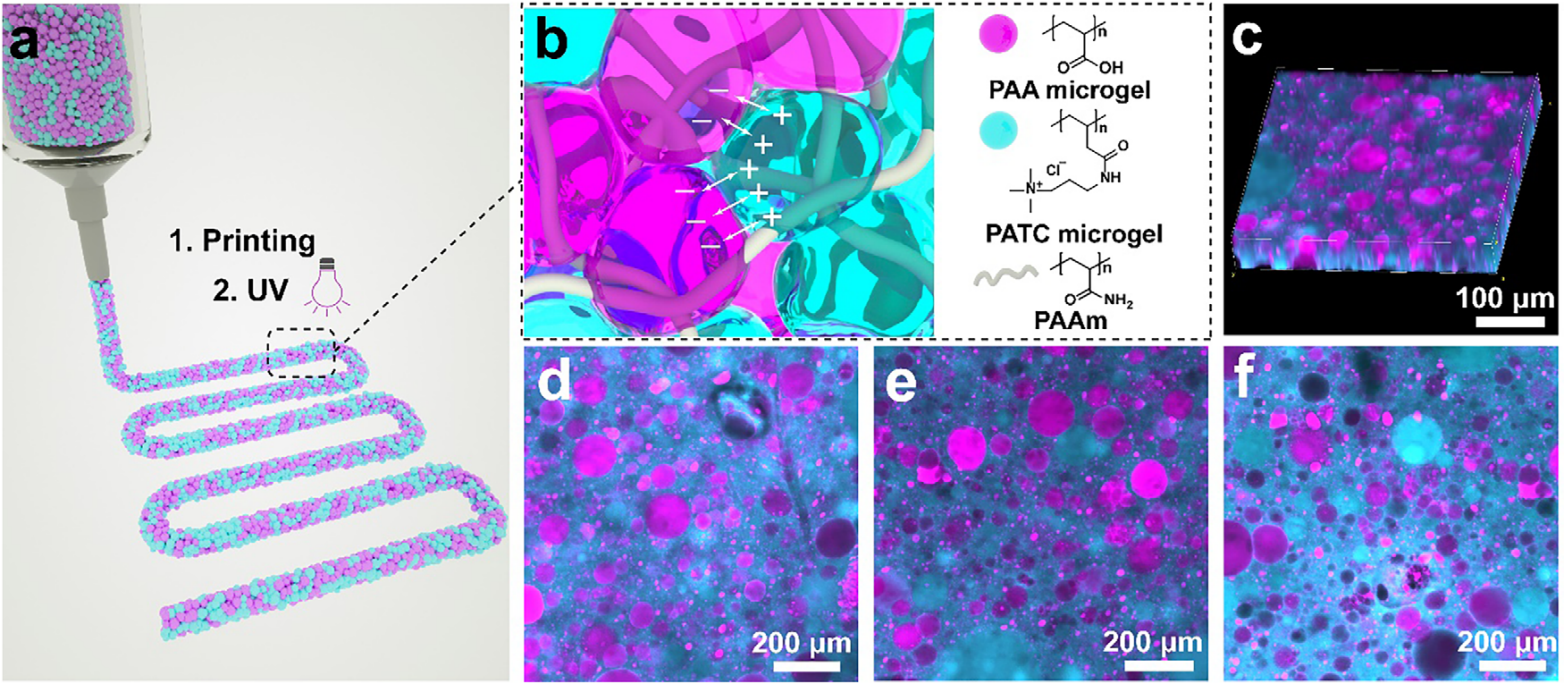
Schematic illustration of electrostatically reinforced DNGHs. a) 3D printing of an ink composed of a mixture of jammed oppositely charged PATC and PAA microgels that have been loaded with AAm through direct ink writing (DIW). b) The 3D‐printed granular structure is rigidified by polymerizing the AAm to form a PAAm network, which interpenetrates the microgels and covalently connects them. c) 3D stack and d–f) individual confocal microscopy images of an interfacially reinforced DNGH reconstructed from confocal microscopy images. PATC microgels are shown in cyan, and PAA microgels in magenta. They are jammed to achieve a total microgel volume fraction of 100%.

Here, we introduce DNGHs possessing an unprecedented fracture energy. These DNGHs are composed of two types of oppositely charged polyelectrolyte microgels, one made of the positively charged poly((3‐acrylamidopropyl)trimethylammonium chloride) (PATC), and the other made of the negatively charged poly(acrylic acid) (PAA). The two oppositely charged microgel types are loaded with water and acrylamide (AAm) precursors, mixed, and jammed to form a 3D‐printable ink, as schematically illustrated in **Figure** **1**a. The granular structure is rigidified by initiating the polymerization of the precursors to yield electrostatically reinforced DNGHs, as shown in Figure 1b and through confocal microscopy in Figure 1c–f. We systematically study the influence of the dissipation zone size, inter‐particle contact area, and adhesion energy on the fracture energy of DNGHs. Based on experimental results, we introduce an empirical model that predicts the fracture energy of this system exclusively using experimentally accessible input parameters. We leverage the electrostatic inter‐particle interactions to 3D‐print centimeter‐sized, free‐standing structures. This material opens up new possibilities for designing strong and tough soft hydrogels, which can be processed into macroscopic materials with intricate 3D structures.

## 2 Results

### 2.1 Influence of Interfacial Reinforcement on the Fracture Energy

We produce spherical microgels from water‐in‐oil emulsion templates. Cationic microgels are produced from a dispersed phase composed of water containing ATC as the monomer and N, N‐methylene bisacrylamide (MBAA) as the cross‐linker. The continuous phase is mineral oil with Abil EM 90 as the surfactant. The phases are emulsified using tip sonication before the photoinitiator Irgacure 1173 is added to the oil, and the drops are converted into microgels by illuminating them with ultraviolet (UV) light. Anionic microgels are produced from a dispersed phase composed of water containing acrylic acid and the photoinitiator. The aqueous phase and mineral oil are mixed at a volume ratio of 1:6 and emulsified. We emulsify the system through vortexing and convert the drops into microgels by exposing them to UV light to initiate the free radical polymerization of the reagents contained in them. The positively charged PATC microgels have a radius of 15.0 ± 9.7 µm, while the negatively charged PAA microgels have a radius of 9.1 ± 3.9 µm, as shown in Figure S1 (Supporting Information). The two types of microgels are dried, and dispersed in water at a well‐defined weight ratio. The microgel mixture is lyophilized. To ensure a microgel volume fraction of 100%, we swell them in the corresponding amount of aqueous solution containing AAm used as a precursor to form the second hydrogel network. This paste is 3D‐printed and solidified by exposing it to UV light to initiate the free radical polymerization of the reagents contained within the microgels. Due to the diffusion of the AAm monomer along its concentration gradient into the interstitial spaces, we expect the network to also form in the interstitial spaces, thereby firmly connecting adjacent microgels, by analogy to what has previously been shown for DNGHs that have not been interfacially reinforced.^[^ ^23^ ^]^

**Figure 2:**
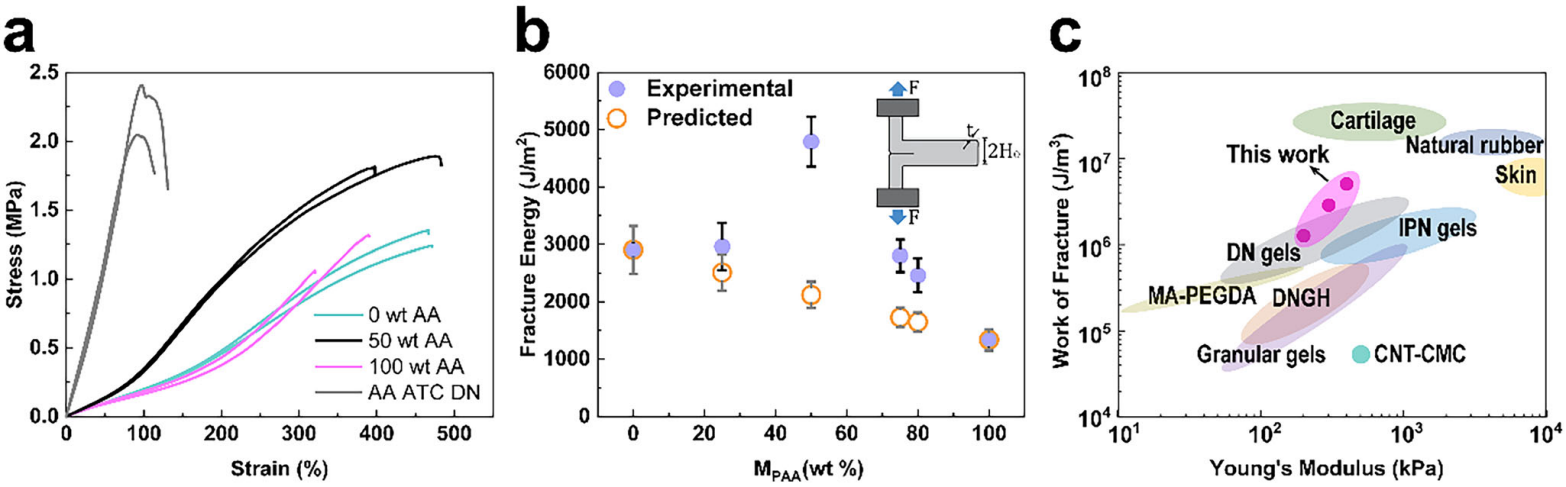
Mechanical properties of interfacially reinforced DNGHs. a) Stress–strain curves of DNGHs composed of a mixture of 50 wt.% PAA and 50 wt.% PATC microgels (black), only PATC microgels (cyan), only PAA microgels (magenta), and a DN hydrogel with the same composition as the DNGHs with mixed microgels (grey). b) Experimentally determined fracture energy and those calculated with the rule of mixture as a function of the weight fraction of PAA microgels in DNGHs containing oppositely charged microgels. Error bars represent standard deviation (SD) with n = 3 for all measurements. The inset illustrates the simple extension test (SET) setup: The undeformed sample is a thin hydrogel slab of length *L*_0_, height 2*H*_0_, and thickness *t*. A long crack of length *c* (*c*  ≫  2*H*_0_) locates in the middle of the sample, and the two legs are clamped on the tensile tester. c) Ashby plot of the work of fracture as a function of the Young's modulus for 3D printable hydrogels, including MA‐PEGDA,^[^ ^32^ ^]^ IPN gels,^[^ ^33^ ^]^ CNT‐CMC,^[^ ^34^ ^]^ granular gels,^[^ ^30^ ^]^ DNGH,^[^ ^13^, ^31^ ^]^ DN,^[^ ^38^ ^]^ and natural materials such as cartilage,^[^ ^27^, ^39^, ^40^ ^]^ skin,^[^ ^29^ ^]^ natural rubbers,^[^ ^27^ ^]^ along with interfacially reinforced DNGHs introduced here (magenta).

To assess the influence of inter‐microgel electrostatic attraction on the mechanical properties of DNGHs, we perform tensile tests as a function of the weight ratio of PATC: PAA microgels within DNGHs. The tensile strength of DNGHs containing 50 wt.% PAA and 50 wt.% PATC microgels is 1.86 ± 0.03 MPa. As shown in **Figure** **2**a, this value is at least 50% higher than that of DNGHs made entirely of either PAA or PATC microgels. This significant enhancement hints at the critical role of interfacial reinforcement in improving tensile strength.

To assess the influence of the microstructure of interfacially reinforced DNGHs on their mechanical properties, we prepare bulk DN hydrogels with the same composition as DNGHs but lacking the granular structure. Bulk DNs are stiffer than DNGHs, as shown in Figure 2a. We assign the increased stiffness of bulk DNs to the structure of the first network, which is continuous in bulk DNs, in stark contrast to that of DNGHs, which is exclusively comprised within the microgels. Since the first network is significantly stiffer than the second network, its discontinuity in DNGHs decreases their Young's modulus. By contrast, bulk DNs are much more brittle than DNGHs, as evidenced by their significantly lower strain at break in Figure 2a. To verify that interfacially reinforced DNGHs are tougher than their DN counterparts, we measure their fracture energy using simple extension tests (SETs), as shown in the experimental setup in the inset of Figure 2b,^[^ ^25^ ^]^ with representative SET curves provided in Figure S2a (Supporting Information). The fracture energy of DNGHs is 4795.4 ± 437.3 J m^−2^, which is nearly four times higher than that of bulk DNs, which is 1202.9 ± 218.2 J m^−^^2^, as shown in Figure S2b (Supporting Information). We assign this difference in fracture energy to the reduced swelling of the first network in bulk DNs due to electrostatic interactions between PATC and PAA.^[^ ^26^ ^]^ This reduction in the degree of swelling decreases the amount of reagents for the second network that can be loaded into the first network and hence, the density of the second network. As a result of the reduced polymer density of the second network, the density of chain entanglements between the first and second networks in bulk DNs is lower than that in DNGHs, ultimately lowering its fracture energy.^[^ ^6^ ^]^

To further confirm that the interfacial attraction between microgels enhances the toughness of DNGHs, we quantify their fracture energy as a function of the weight fraction of PAA microgels. These experimentally determined values are compared to estimates calculated using the rule of mixture based on the fracture energy of DNGHs composed solely of PAA or PATC microgels. All DNGHs containing oppositely charged microgels exhibit higher fracture energies than the values predicted by the rule of mixture, as shown in Figure 2b. For instance, the measured fracture energy of DNGHs containing 50 wt.% PAA microgels is nearly double the value calculated with the rule of mixture, indicating that interfacial reinforcement strongly contributes to the fracture energy of this system. Indeed, by tuning the weight fraction of PAA microgels contained within these DNGHs, their fracture energy can be varied from 904 to 4795 J m^−2^, the work of fracture can be changed from 1.12 to 5.11 MJ m^−3^, and the Young's modulus varies from 201 to 307 kPa. Notably, their work of fracture is similar to those found in skin and natural rubber,^[^ ^27^, ^28^, ^29^ ^]^ and surpasses values reported for direct ink written hydrogels,^[^ ^13^, ^30^, ^31^, ^32^, ^33^, ^34^ ^]^ as illustrated in the Ashby plot Figure 2c.

**Figure 3:**
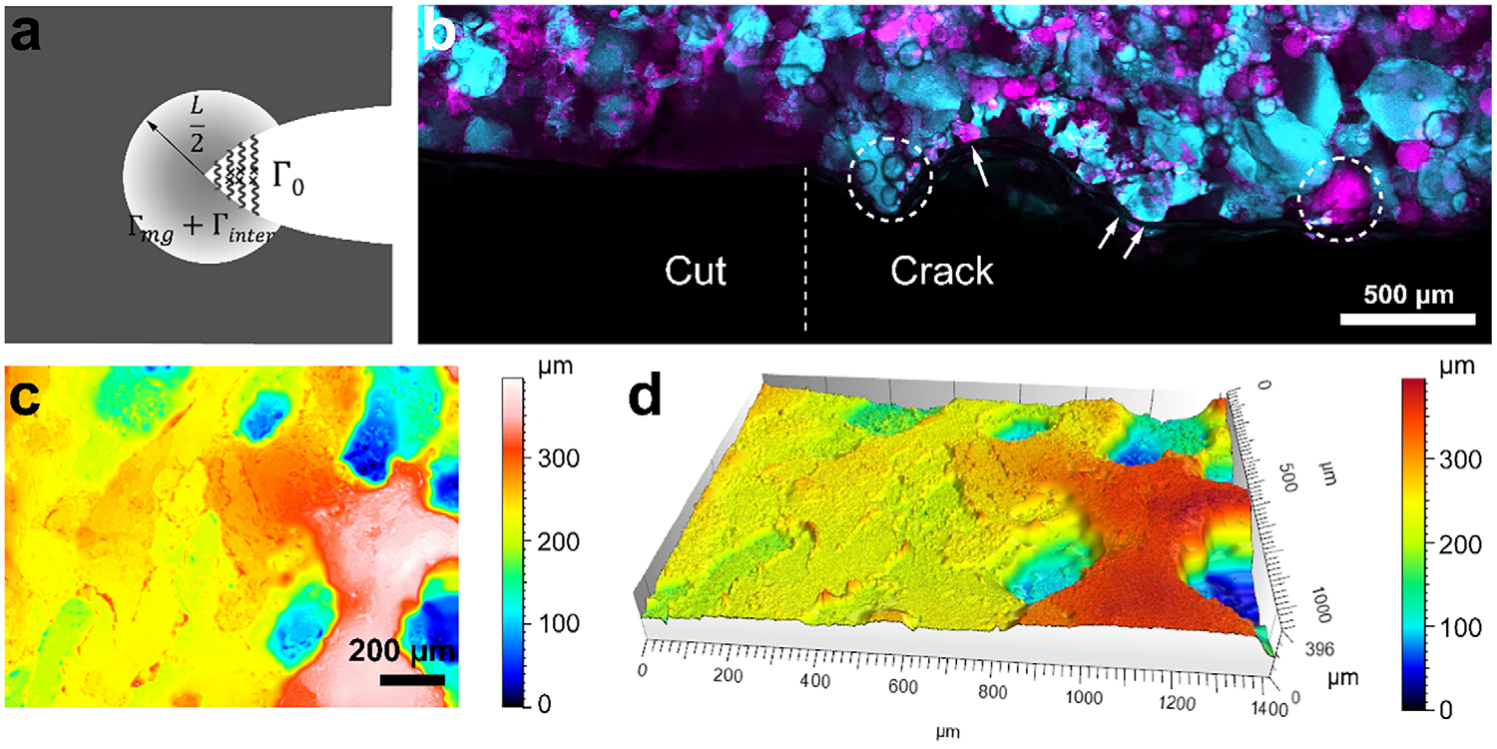
Schematic illustration of the different components influencing the fracture energy Γ of interfacially reinforced DNGHs. a) The fracture energy Γ is composed of the intrinsic fracture energy Γ_0_, dissipated fracture energy Γ_mg_, and Γ_inter_ in the dissipation zone with a radius of $\frac{L}{2}$. b) Confocal microscopy image of a fractured DNGH. The initial crack, created using a razor blade, is located to the left of the dashed line, while the propagated crack is observed on the right. During propagation, the crack primarily advances around the microgels (indicated by circles) although it sometimes also crosses microgels (highlighted by arrows) c) 2D profile, and d) 3D reconstruction of the crack morphology in the fractured DNGH.

To estimate the contribution of the interparticle electrostatic interaction to the overall fracture energy of DNGHs, we introduce an empirical equation based on an equation that describes the fracture energy of bulk DNs.^[^ ^35^ ^]^ The fracture energy Γ of bulk DNs is the sum of the intrinsic fracture energy Γ_0_ and the dissipated fracture energy Γ_*d*_. By analogy, we propose that the fracture energy Γ of DNGHs is the sum of three contributions, as illustrated in **Figure** **3**a: the intrinsic fracture energy Γ_0_ of the second network, the fracture energy dissipated within the damaged microgels Γ_*mg*_, and the fracture energy dissipated between the microgels Γ_inter_.

(1) $$\Gamma =\Gamma_{0}+\Gamma_{\mathrm{mg}}+\Gamma_{\mathrm{inter}}$$

The intrinsic fracture energy Γ_0_ is associated with the scission of polymer chains in the second network. The dissipated fracture energy Γ_mg_ is due to polymer chain scissions within the microgels located in the dissipation zone around the crack tip. To account for the electrostatic attraction between oppositely charged particles, we introduce Γ_inter_.

The contribution of the energy required to dissipate inter‐particle electrostatic attraction forces to the total fracture energy can only be verified if the crack mainly propagates within the interstitial spaces. To assess if this is the case, we perform post‐mortem confocal microscopy to image the propagated crack in the fractured DNGH. Indeed, microgels tend to deflect the crack, such that it primarily propagates within the interstitial space although occasionally, it also propagates across microgels, as shown in Figure 3b. Surface roughness measurements performed on the crack plane confirm that microgels deflect the propagating cracks, as illustrated in Figure 3c,d. Compared to the smooth cut surface created by a razor blade in Figure S3 (Supporting Information), the propagated crack is much rougher. The average roughness *Sa* of the propagated crack is 47.3 µm, which is five times greater than that of the razor‐cut surface, which is 9.0 µm. This crack deflection increases the crack front complexity and further enhances the fracture energy of DNGHs.^[^ ^36^, ^37^ ^]^

To quantify the contribution of Γ_inter_, we first quantify Γ by performing SET on DNGHs. Similarly, Γ_0_ is measured by performing SET on PAAm bulk hydrogels possessing the same composition as the second network in DNGHs, as demonstrated in Figure S4 (Supporting Information). The electrostatic reinforcement does not appreciably affect the dissipative length scales, as summarized in Table S1 (Supporting Information). Hence, we calculate Γ_mg_ from DNGHs composed solely of either PATC or PAA microgels whose Γ_inter_ = 0. We do so by subtracting Γ_0_ from the corresponding Γ. Using these values, Γ_inter_ is calculated as Γ_inter_ = Γ − Γ_0_ − Γ_mg_, as detailed in Figure S5 (Supporting Information). We propose an empirical equation to estimate the dissipated fracture energy, Γ_inter_′:

(2) $${\Gamma_{\mathrm{inter}}}^{\prime }=\frac{S}{V}\times E_{a}\times \frac{L}{2}$$

Here, $\frac{S}{V}$ represents the surface‐to‐volume ratio, where *S* is the contact area between the oppositely charged microgels, and *V* the volume of the region of interest. *E*_a_ denotes the adhesion energy, defined as the energy required per unit area to separate the oppositely charged microgels. *L* corresponds to the dissipation zone size. A detailed derivation of this equation is provided in the Supporting Information. To validate this empirical equation, we experimentally vary $\frac{L}{2}$, $\frac{S}{V}$, and *E_a_*, and compare the values of Γ_inter_′ with Γ_inter_ determined by SET.

### 2.2 Quantification of the Dissipation Zone Size *L*

**Figure 4:**
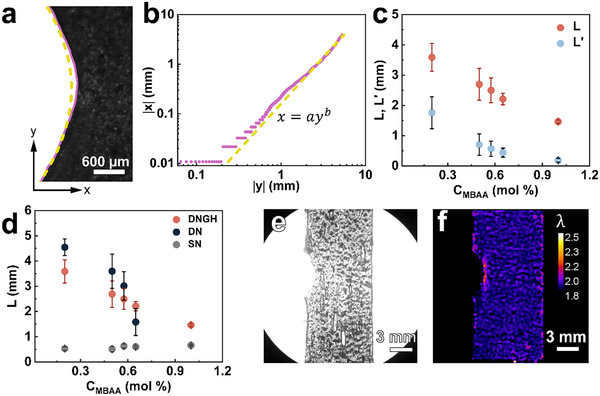
Quantification of the dissipation zone size of DNGHs made of 50 wt.% PAA and 50 wt.% PATC microgels that are connected by a second network containing 0.2 mol % of MBAA. a) The optical microscopy image of a crack tip. The curve of the crack is extracted using the Python Skimage package and indicated by a solid magenta line.^[^ ^50^ ^]^ The extracted curve is fitted to a power law *x*  =  *ay^b^*, where *x* and *y* are the coordinates of the point on the curve. The fitted curve is indicated by a yellow dashed line. b) The fitted curve starts to deviate from CTOD at |*x*| =  0.4 mm and |*y*| =  2 mm. c) Comparison between the dynamic length scale *L* (red) and the energy dissipation length scale *L*’ (blue). d) Influence of the cross‐linker concentration in the second network, *C_MBAA_*, on the dissipation zone size *L* of electrostatically reinforced DNGHs (red), PATC‐PAAm bulk DNs (blue), and a single network of PAAm (grey). e) Optical microscopy image of the crack propagation within a DNGH containing a second network with *C_MBAA_* = 0.2 mol %, and its corresponding f) maximum principal stretch determined by DIC. A highly stretched zone is observed at the crack tip.

In bulk DN hydrogels, the dissipated fracture energy, Γ_d_, depends on the size of the dissipation zone *L*.^[^ ^35^ ^]^ To assess if this also applies to DNGHs, we quantify *L* by conducting crack tip opening displacement (CTOD) measurements, following established procedures for tough bulk DN hydrogels and brittle single network hydrogels.^[^ ^24^, ^37^ ^]^ We visualize the crack propagation with time‐lapse optical microscopy and fit the CTOD to a power law *x*  =  *ay^b^*, as exemplified in **Figure** **4**a. The CTOD measured on DNGHs containing 50 wt.% PAA microgels and 50 wt.% PATC microgels only starts to deviate from the fitted curve around the crack tip, as quantified in Figure 4b. These results indicate that the dissipation zone is localized at the crack tip.^[^ ^41^ ^]^ We fit this curve to obtain values for *a* and *b*, as detailed in Figure S6 (Supporting Information). According to linear elastic fracture mechanics (LEFM), the stress σ at a distance *r* from the crack tip scales as $\sigma \;\approx \;1/\sqrt{r}$.^[^ ^42^, ^43^ ^]^ This scaling results in a parabolic crack opening profile such that the exponent *b* in the CTOD should be 2 for linear elastic materials that undergo small strains during crack propagation. However, the *b* values of DNGHs are below 2, indicating that interfacially reinforced DNGHs undergo large strains during crack propagation that involve nonlinear effects.^[^ ^24^ ^]^ To account for this behavior, we quantify the dissipation zone size *L* using a dynamic length scale defined by a non‐trivial power law $L=a^{\frac{1}{b-1}}\;$, which is appropriate for tough gels capable of sustaining large strains before failure.^[^ ^24^ ^]^ To test whether this dynamic length scale is proportional to the energy dissipation length scale at the crack tip, we define a naïve energy dissipation length scale, *L*′, calculated from Equation 2. Indeed, *L* and *L*′ are propotional even though *L* is larger than *L*′, as shown in Figure 4c. This discrepancy in absolute values likely arises from our simplified assumption that the energy dissipation is uniform across the dissipation zone.^[^ ^44^, ^45^ ^]^ Based on this analysis, we use *L* as an estimation of the dissipation zone size. These measurements reveal a dissipation zone size in the millimeter range, a value similar to those reported for tough bulk DN hydrogels.^[^ ^7^, ^35^, ^46^ ^]^

The dissipation zone size of bulk DNs depends on their threshold stretch, where the crack propagation begins.^[^ ^46^ ^]^ This value is influenced by the time‐dependent behavior of the secondary network,^[^ ^46^ ^]^ which depends on its viscoelastic behavior and can be adjusted with its cross‐link density.^[^ ^47^ ^]^ To assess if this is also the case for electrostatically reinforced DNGHs, we vary the strain at break of the second network by adjusting the cross‐linker concentration. Indeed, the dissipation zone size increases with decreasing cross‐linker concentration C_MBAA_ in the second network, reaching a maximum of 3.6 ± 0.5 mm for a C_MBAA_ of 0.2 mol%, as shown in Figure 4d. This damage zone size is more similar to that of bulk PATC‐PAAm DN hydrogels than that of the single PAAm network, suggesting that the energy dissipation mechanisms in our DNGHs resemble those of bulk DN hydrogels. We are unable to reduce the MBAA concentrations below 0.2 mol%, as lower MBAA concentrations compromise the integrity of the DNGHs. Higher MBAA concentrations result in stiffer second networks that display smaller dissipation zone sizes, as shown in Figure 4d. Hence, we fix the cross‐linker concentration, C_MBAA_, in the second network to 0.2 mol% for all subsequent experiments, unless specified otherwise.

The dissipation zone size is related to the non‐linear deformation zone at the crack tip.^[^ ^48^ ^]^ Our results suggest that electrostatically reinforced DNGHs possess a large dissipation zone, where we anticipate a non‐linear deformation at the crack tip. To visualize this non‐linear deformation zone in situ, we calculate the strain field of the inelastic zone at the crack tip using digital image correlation (DIC) performed on time‐lapse optical microscopy images, shown in Figure 4e. Indeed, the region at the crack tip is more strained than areas located further away from the crack tip, as shown in Figure 4f. This observation is supported by the principal stretch profile presented in Figure S7 (Supporting Information). This result suggests that within the strained zone at the crack tip, DNGHs are more damaged and consequently dissipate more energy than in the areas outside this zone. The length of this strained zone is in the millimeter range, which is consistent with the value estimated by CTOD. Note that the correlation between the dissipation zone size and the length of the deformation zone at the crack tip is unclear, which limits our ability to quantitatively confirm the dissipation zone size from these measurements. However, it can be qualitatively assessed, as the deformation zone is closely related to the dissipative zone at the crack tip.^[^ ^49^ ^]^

### 2.3 Quantification of the Interparticle Contact Area $\frac{S}{V}$

**Figure 5:**
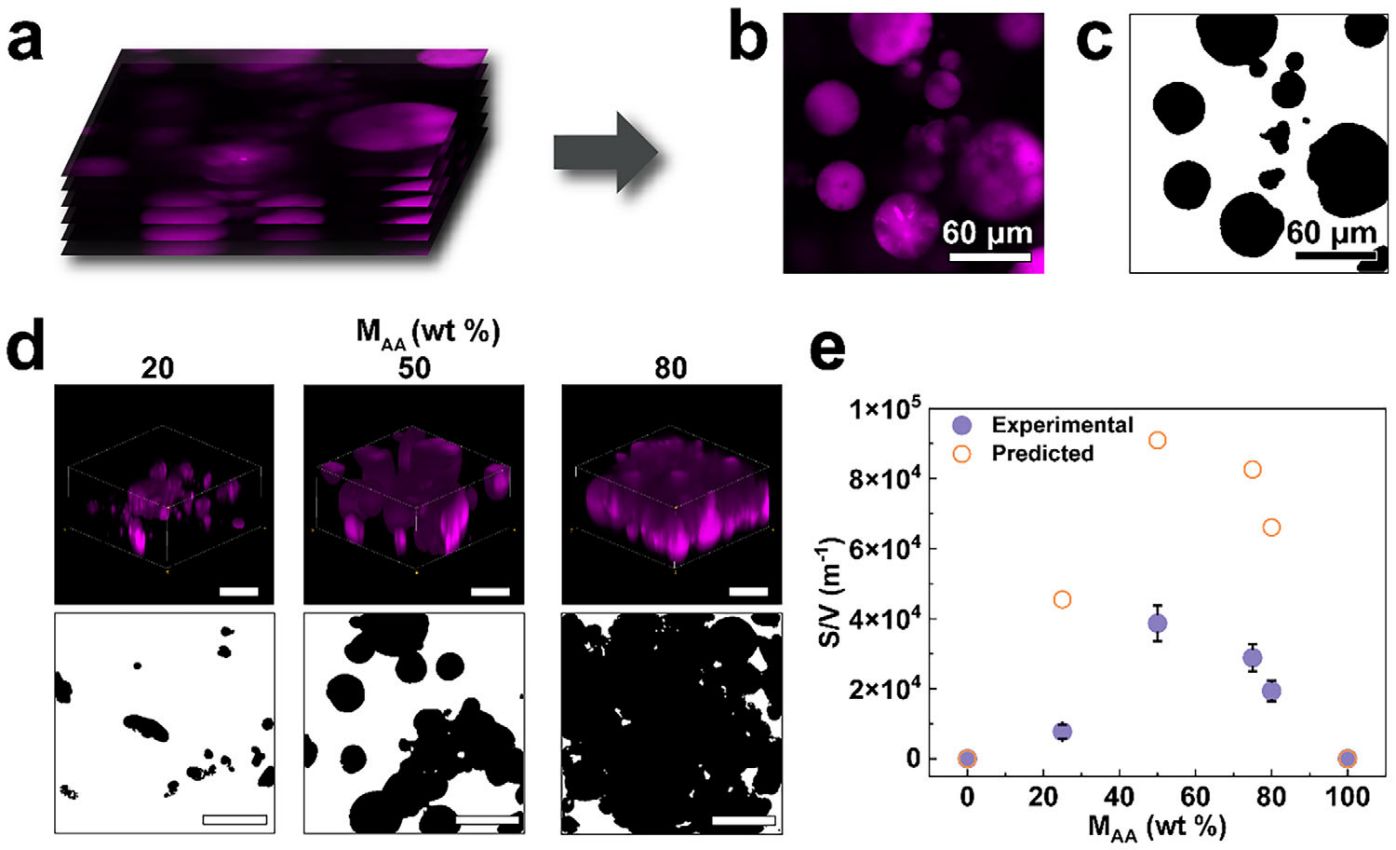
Quantification of the contact area between oppositely charged microgels. a) Z‐stack confocal images of a DNGH containing 50 wt% PAA microgels and 50 wt.% PATC microgels, marked in magenta. b) Each slice is c) thresholded to calculate the contact area between PAA and PATC microgels. d) 3D reconstruction (top) and thresholded slices (bottom) of DNGHs containing 20, 50, and 80 wt.% of PAA microgels. Scale bars are 60 µm. e) Comparison between the experimental $\frac{S}{V}$ and that calculated from the average microgel size, assuming perfectly spherical microgels as a function of the weight fraction of *M_PAA_*.

Our results suggest that the weight ratio of the two types of oppositely charged microgels within our DNGHs strongly influences their fracture energy. To assess if this correlation is related to the contact area *S* between oppositely charged microgels, we quantify this parameter and normalize it by the analyzed volume *V* within DNGHs to obtain the surface‐to‐volume ratio, $\frac{S}{V}$. To distinguish the two types of microgels with confocal microscopy, we label PAA microgels with Rhodamine B (Rho B). We acquire Z‐stacks and use them to calculate $\frac{S}{V}$ as a function of the weight fraction of PAA microgels contained in DNGHs. Each slice in the Z‐stack is thresholded based on a greyscale value between 50 and 255, as shown in **Figure** **5**a–c and detailed in Figure S8 (Supporting Information). As expected, the total microgel volume fraction is 100%, as shown in Figure 1d–f. Hence, we calculate $\frac{S}{V}$ by quantifying the perimeter of the thresholded PAA microgels in close contact with PATC microgels, multiplying this value by the Z‐stack height, and dividing it by the total volume of the stack. Indeed, the volume normalized contact area between oppositely charged microgel peaks at a PAA microgel fraction of 50 wt.%, as qualitatively shown in the confocal microscopy images in Figure 5d and summarized in Figure 5e. To further validate our measurements, we calculate the theoretical contact area based on the average microgel sizes and their weight fractions, as shown in Figure 5e. The calculated values are consistently higher than the measured ones. We assign this discrepancy to the fact that in the calculations, we assume each particle is fully surrounded by oppositely charged particles. However, our confocal microscopy images reveal the presence of some agglomerates, which significantly reduce $\frac{S}{V}$. Despite this discrepancy, the trends are consistent.

### 2.4 Quantification of the Adhesion Energy *E_a_*

**Figure 6:**
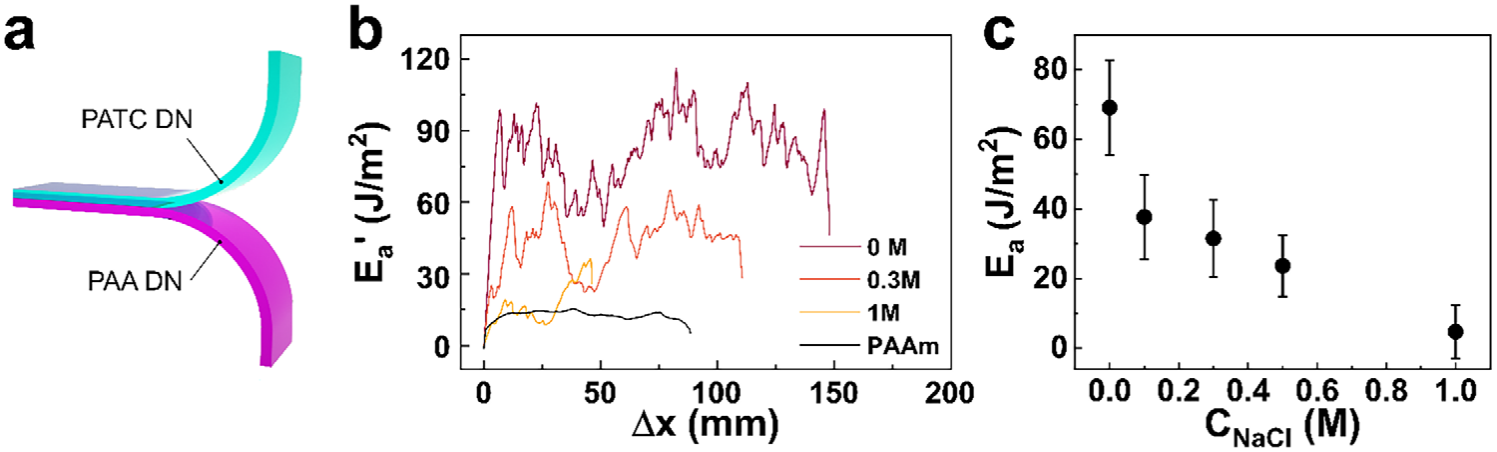
Quantification of the adhesion energy between PAA and PATC microgels E_*a*_. a) Schematic illustration of a 180° peel test on bulk slabs composed of a PATC/PAAm DN and a PAA/PAAm DN hydrogel. b) Representative curves of peel tests performed on bulk PAA and PATC DN hydrogel slabs as a function of the NaCl concentration contained in them. c) Averaged plateau values of the adhesion energy, E_*a*_, measured in (b) as a function of the NaCl concentration contained in the hydrogels, *C*_NaCl_.

The fracture energy of our DNGHs significantly increases with the contact area of oppositely charged microgels. This correlation suggests that the adhesion energy between oppositely charged microgels influences the fracture energy of DNGHs. To estimate the adhesion energy between PATC and PAA microgels, we quantify the adhesion energy between bulk PAA/PAAm and PATC/PAAm double network hydrogels using 180° peel tests, as shown in **Figure** **6**a. We determine the adhesion energy E_*a*_′ as the plateau value of this curve, which reveals E_*a*_′ = 82.3 ± 13.6 J m^−2^. To verify that this adhesion energy is indeed associated with the electrostatic attraction between the two oppositely charged polyelectrolytes, we quantify E_*a*_′ as a function of the Sodium Chloride (NaCl) concentration contained within the gels. As expected, the adhesion energy between the two oppositely charged hydrogel slabs decreases from 82.3 to 18.0 ± 8.0 J m^−2^ if we increase the NaCl concentration from 0 to 1 M, as shown in Figure 6b. We assign the decrease in the adhesion energy to an ion‐induced partial screening of the electrostatic interactions.^[^ ^51^ ^]^ This result confirms that the interfacial adhesion is caused by an electrostatic attraction of oppositely charged microgels. To exclude any effect of PAAm on the adhesion energy between PATC and PAA, we measure E_*a*_′ between two single network slabs composed of PAAm, which is 13.3 ± 1.1 J m^−2^, as shown in Figure 6b. We subtract this value from E_*a*_′ between PAA and PATC DN, yielding a maximum electrostatic attraction‐based adhesion energy E_*a*_ of 69.1 ± 13.6 J m^−2,^ as summarized in Figure 6c.

### 2.5 The Influence of *L*, $\frac{S}{V}$, and *E*_a_ on the Fracture Energy of DNGHs

**Figure 7:**
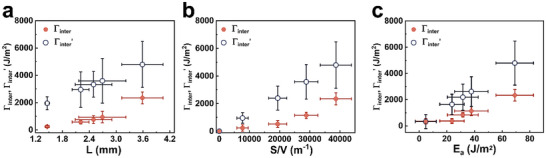
Calculated Γ_inter_′ and experimentally measured Γ_inter_ as a function of the a) dissipation zone size *L*, b) surface‐to‐volume ratio $\frac{S}{V}$, and c) adhesion energy E_a_.

The empirical model introduced as Equation (2) predicts the fracture energy of DNGHs using the experimentally accessible input parameters *L*, $\frac{S}{V}$, and *E_a_*. This model predicts a maximum Γ_inter_′ = 4791.6 ± 1678.8 J m^−2^ for DNGHs containing 50 wt.% PATC and 50 wt.% PAA microgels. To experimentally assess the validity of this model, we quantify Γ_inter_ as a function of *L*, $\frac{S}{V}$, and *E*_a_ with SET, as detailed in Figures S9–S11 (Supporting Information). We observe similar trends between Γ_inter_ and Γ_inter_′, although the predicted Γ_inter_′ is consistently higher than Γ_inter_ measured from SET, as shown in **Figure** **7**. We attribute this discrepancy to gradients of the dissipated energy density across the dissipation zone, which we neglected in our estimation. Nevertheless, our results suggest that Γ_inter_, and consequently the fracture energy Γ can be estimated using the empirical model. Remarkably, Γ_inter_ accounts for up to 49% of the overall fracture energy Γ. The significant contribution of Γ_inter_ to the overall fracture energy enables tuning of this parameter. Γ_inter_ can be tuned by adjusting *L* through the crosslink density of the second network, $\frac{S}{V}$ with the weight ratio between oppositely charged microgels contained within DNGHs, and *E*_a_ by altering the salt concentration in DNGHs.

### 2.6 3D‐Printing of Interfacially Reinforced DNGHs

**Figure 8:**
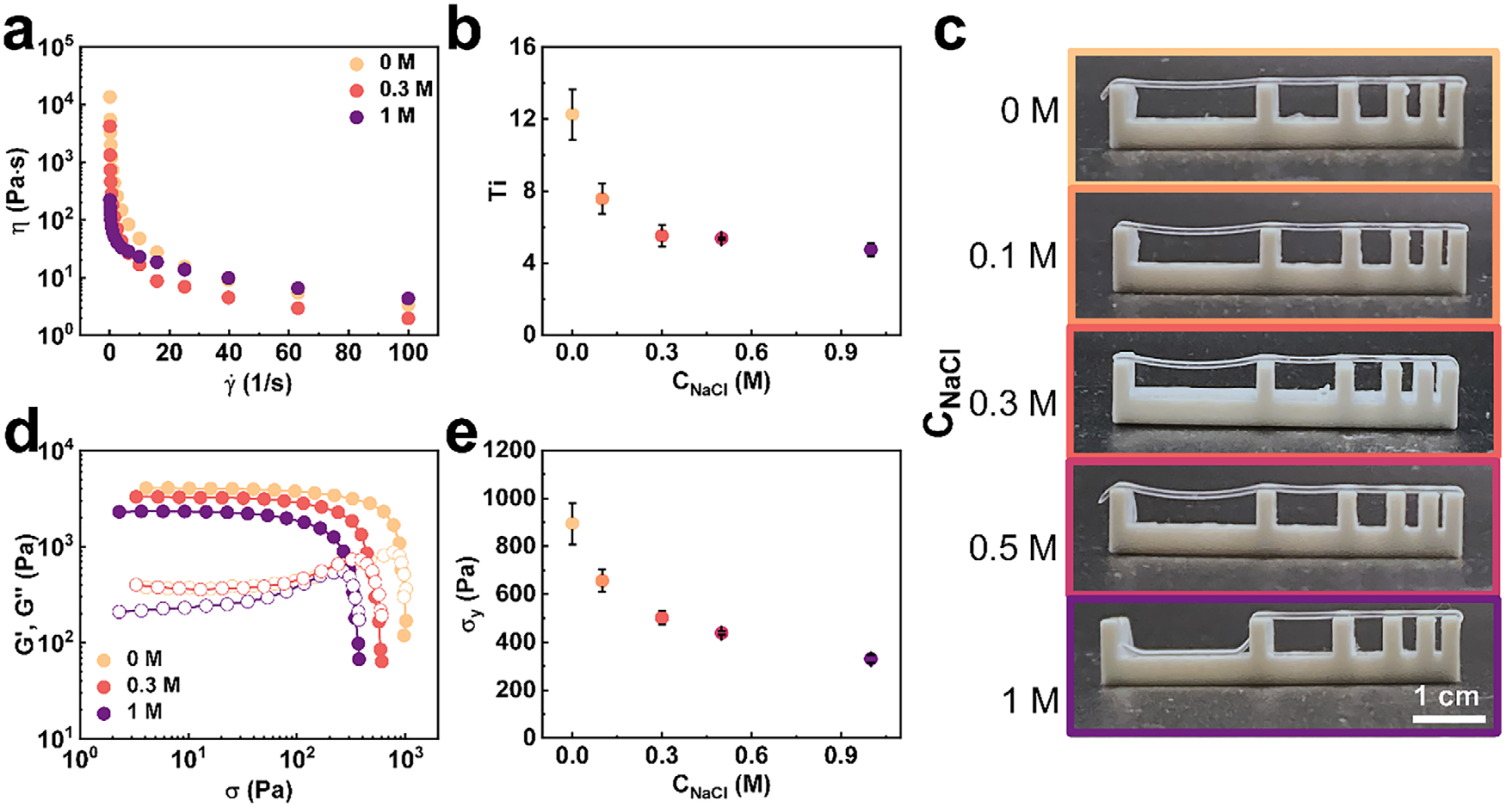
Shape retention and rheological properties of 3D printed inks. a) Frequency‐dependent viscosity of inks, composed of 50 wt.% PATC and 50 wt.% PAA microgels. Microgels have been soaked in an aqueous solution containing 40 wt.% AAm and 0 m (yellow), 0.3 m (red), and 1 m NaCl (purple). b) The thixotropic index of the inks as a function of the NaCl concentration. c) Photographs of 3D printed PATC and PAA microgel filaments deposited onto supporting pillars as a function of the NaCl content within them. The distances between pillars are 16, 8, 4, 2, and 1 mm and the pillar width is 2 mm. d) Amplitude sweep of inks composed of 50 wt.% PATC and 50 wt.% PAA microgels, soaked in an aqueous solution containing 40 wt.% AAm and NaCl. The yield stress of the ink is determined at the crossover between the storage modulus G' (filled circle) and the loss modulus G'' (empty circle). e) The yield stress σ_y_ of microgel pastes as a function of the NaCl concentration in the aqueous solution they have been swollen in.

To demonstrate the potential of electrostatically reinforced DNGHs to be printed into centimeter‐sized free‐standing structures, we process them through DIW. DIW imparts stringent rheological properties, such as shear thinning, a low yield point, and fast stress recovery to its inks.^[^ ^52^ ^]^ To assess the influence of the inter‐microgel electrostatic interactions on the rheological properties of inks composed of jammed microgels, we perform oscillatory rheology on them. All tested inks are shear‐thinning, independent of the NaCl concentration contained within the gels, as shown in **Figure** **8**a. Yet, the viscosity at low shear rates increases with decreasing NaCl concentration, which indicates that the electrostatic interactions influence the static mechanical properties more strongly than the dynamic ones. This behavior can be attributed to the dissociation of electrostatic interactions between microgels under high shear rates, due to increased inter‐particle distances.^[^ ^14^ ^]^ To assess the effect of the NaCl concentration on ink printability, we quantify the thixotropic index (*Ti*). The thixotropic index reflects the ability of the ink to flow during extrusion and its structural integrity upon removal of the shear.^[^ ^53^, ^54^ ^]^ *Ti* is calculated as:

(3) $$Ti=\frac{\eta_{\gamma 10}}{\eta_{\gamma 100}}\;$$

where η_γ10_ is the viscosity at a shear rate of 10 s^−1^ and  η_γ100_ is the viscosity at a shear rate of 100 s^−1^. The thixotropic index increases with decreasing NaCl concentration in the ink, as shown in Figure 8b. These results indicate that lower NaCl concentrations enhance the structural integrity of the ink, rendering it more suitable for 3D printing. A high thixotropic index improves the shape retention of the ink.^[^ ^53^, ^54^ ^]^ To highlight the significance of this effect, we perform filament collapse tests.^[^ ^55^ ^]^ For a fixed gap size, the filament sagging decreases with decreasing NaCl concentration, as shown in Figure 8c. Remarkably, in the absence of salts, an ink composed of 50 wt.% PATC and 50 wt.% PAA microgels does not significantly sag even if the distance between pillars is as long as 16 mm. This result suggests that the yield stress of this filament, σ_*y*_, estimated as $\frac{\rho gL}{2}$, must exceed 80 Pa; here, ρ denotes the density of the ink, *g* the gravitational acceleration, and *L* the distance between the pillars. To verify this indication and further quantify the effect of the interparticle interactions on σ_*y*_, we conduct stress sweeps on inks. Indeed, the yield stress of the inks increases from 330.6 ± 15.6 to 894.7 ± 86.2 Pa if *C*_NaCl_ decreases from 1 to 0 m, as shown in Figure 8d,e. These high‐yield stresses can improve the shape retention of the ink.

**Figure 9:**
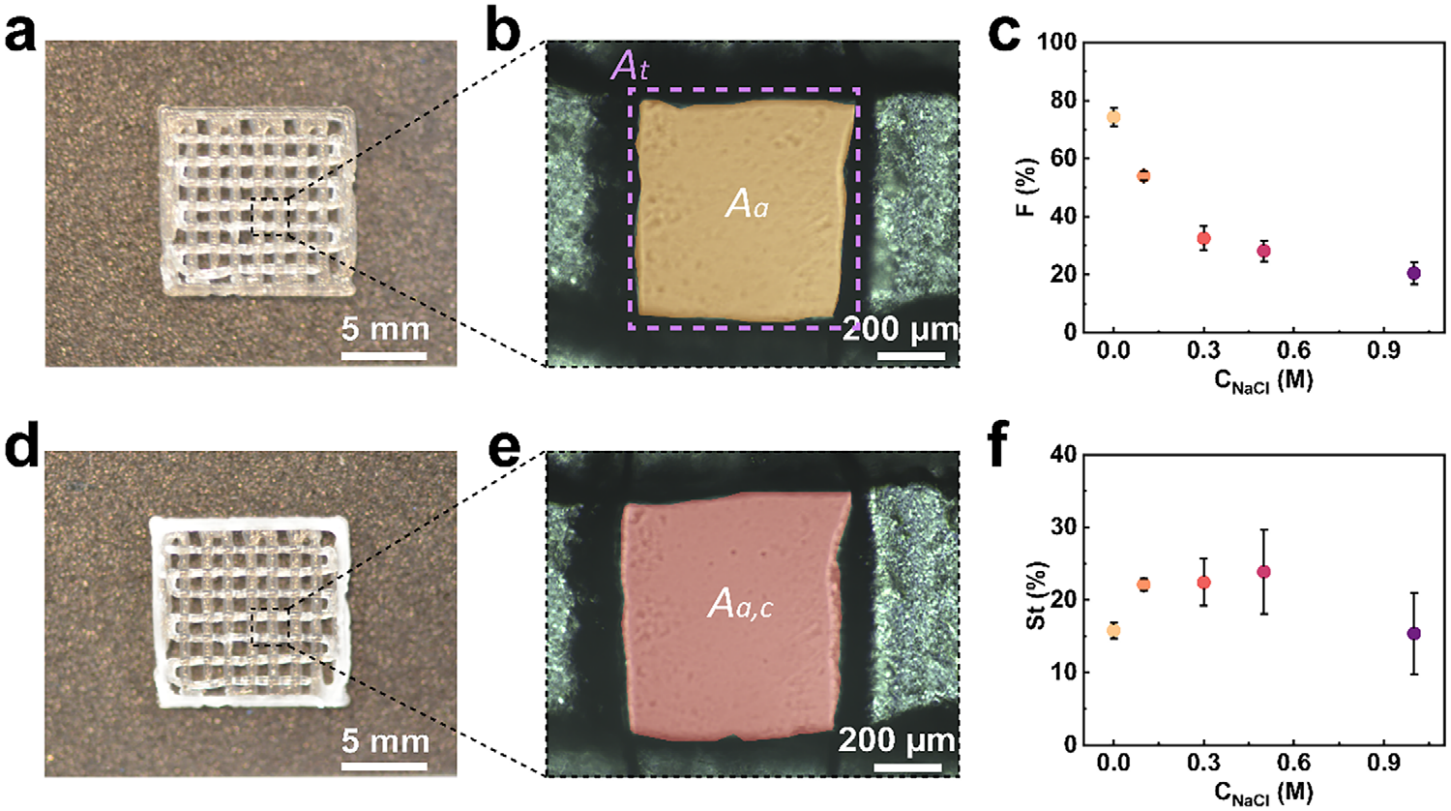
Printing resolution and shape stability of 3D printed inks. a) Photograph of an as‐printed lattice using an ink composed of 50 wt.% PATC and 50 wt.% PAA microgels, and 40 wt% AAm. b) Optical micrograph of a cell within the lattice. The orange‐shaded area is the actual area *A_a_* of the cell, whereas the dashed box represents the theoretical area *A_t_*. c) The shape fidelity F of inks as a function of the NaCl concentration within them. d) Photograph of the polymerized lattice printed in (a). e) Optical micrograph of the same cell in (b) after polymerization. f) The shape stability *St* of the inks after polymerization as a function of the NaCl concentration in the ink. The calculation of shape fidelity and shape stability is detailed in the experimental methods.

To assess the influence of the NaCl concentration on the printing resolution, we print lattices with a dimension of 10 mm × 10 mm and quantify the shape fidelity *F*, as exemplified in **Figure** **9**a,b, and in Figure S12a,b (Supporting Information). The shape fidelity increases from 20.5 ± 3.7% to 74.3 ± 3.2% if the NaCl concentration decreases from 1 to 0 M, as shown in Figure 9c. This finding demonstrates that the electrostatic attraction of oppositely charged microgels enhances printing resolution. We also quantify the shape stability, *St*, of the ink before and after the photo‐polymerization, as demonstrated in Figure 9d,e, and Figure S12c,d (Supporting Information). The objects shrink by ≈20% upon photo‐polymerization, as shown in Figure 9f. We assign the reduction in object size to a partial drying that occurs during the polymerization process.

**Figure 10:**
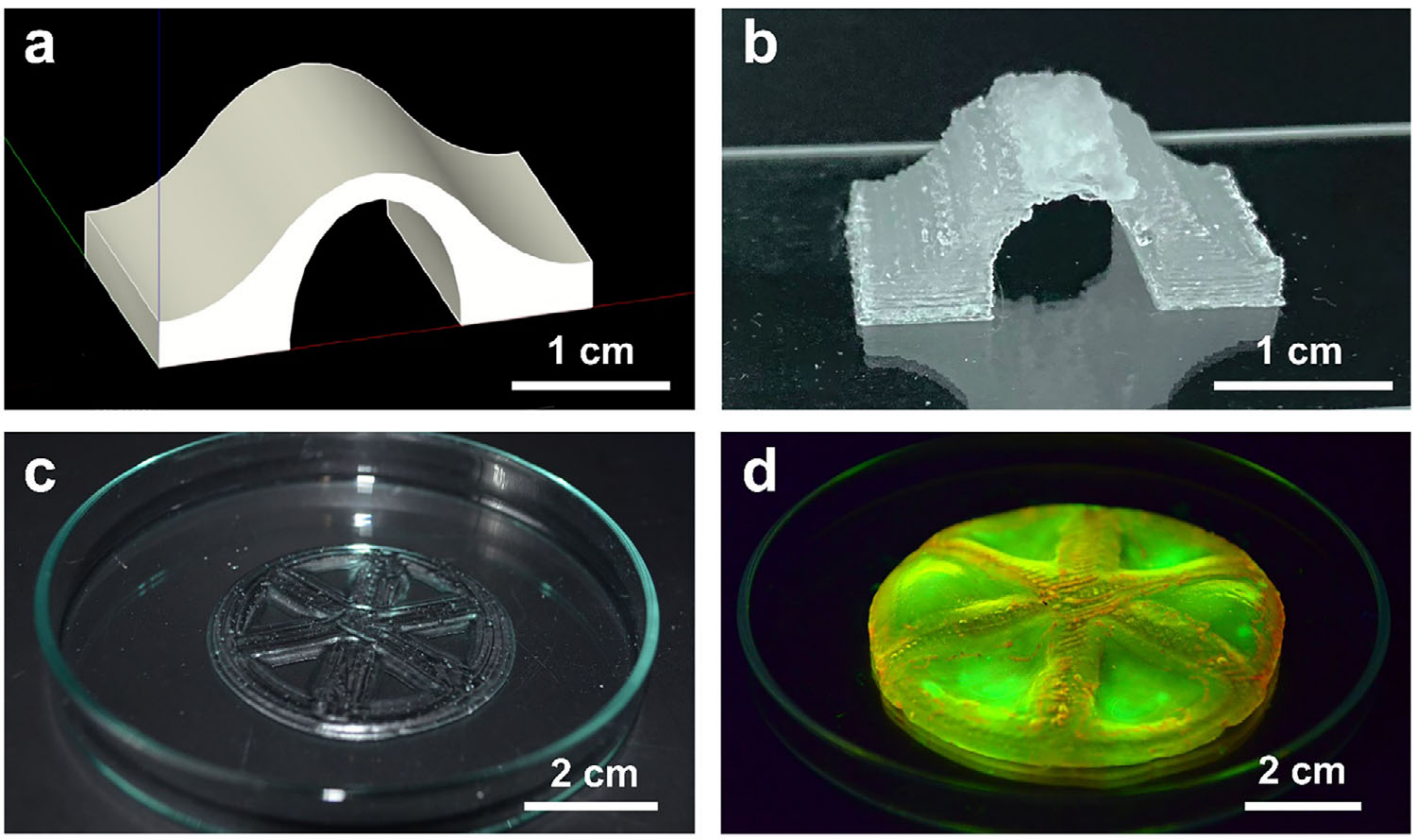
3D printing of interfacially reinforced DNGHs. a) The original 3D design and b) photograph of the 3D printed bridge composed of 26 layers of DNGHs containing 50 wt.% PAA microgels. c,d) Photographs of a wheel that has been 3D printed from two types of inks (c) before and (d) after it has been swollen in an aqueous solution. The frame and ridges of the wheel are composed of a DNGH containing a mixture of 50 wt.% PAA and 50 wt.% PATC microgels, and the spoke gap is made from a DNGH containing exclusively 50 wt.% PATC microgels. For visualization, the spokes and rim are stained by swelling the polymerized structure in an aqueous solution containing Rho B that selectively binds to PAA microgels, and the spoke gap with fluorescein, which selectively binds to PATC microgels.

To demonstrate the potential of our material to be processed into intricate 3D structures, we print a suspending bridge without any supporting structures or baths, as exemplified in **Figure** **10**a,b. Structures possessing locally varying compositions can also be achieved by co‐printing a rigid ink containing a mixture of 50 wt.% PATC and 50 wt.% PAA microgels with a soft ink only composed of 50 wt.% PATC microgels. Because of confinement reasons, the degree of swelling of DNGHs increases with decreasing amount of microgels contained in them.^[^ ^23^ ^]^ We exploit this feature to 3D‐print shape morphing structures. We 3D‐print a wheel with a rim and spokes composed of the rigid ink and spoke gaps made of the soft ink, as shown in Figure 10c. If immersed in an aqueous solution, the soft DNGH swells more than the rigid one. Yet, this expansion is limited by the surrounding rim such that the soft DNGH starts to bulge, as shown in Figure 10d.

## 3 Conclusion

We introduce a 3D‐printable interfacially reinforced DNGH displaying an unprecedented fracture energy. This is achieved by leveraging electrostatic attraction forces between adjacent microgels. We demonstrate that the energy required to separate oppositely charged microgels contained within DNGHs contributes up to 49% of their total fracture energy. The energy required to dissipate oppositely charged microgels within DNGHs depends on the dissipation zone size, the contact area between oppositely charged microgels, and the adhesion energy. Although the correlation is qualitative, it provides a framework for effectively tuning the fracture energy of electrostatically reinforced DNGHs through experimentally accessible parameters. For example, the interfacial reinforcement can be tuned with the crosslinker concentration within the second network, the weight ratio between oppositely charged microgels, and the salt concentration contained in the hydrogel. The inter‐particle electrostatic attraction offers another advantage: it increases the zero shear viscosity of the system, thereby significantly improving its shape retention and facilitating the printing of free‐standing structures. By tailoring the ink composition, we envisage this ink to enable the fabrication of adaptive implants capable of dynamically changing their morphology in response to changes in the environment^[^ ^56^, ^57^ ^]^ Moreover, its stability in aqueous environments renders it promising for underwater shock absorbers.^[^ ^58^, ^59^ ^]^

## 4 Experimental Section

### Synthesis of PAA Microgels

Microgels are produced from water‐in‐oil emulsion templates. The aqueous phase contains 30 wt.% acrylic acid (AA) (Sigma–Aldrich, USA), 3.5 mol % N, N‐methylene bisacrylamide (MBAA) (Carl Roth, Germany), and 5 µL mL^−1^ 2‐hydroxy‐2‐methylpropriophenone (Irgacure 1173) (Sigma–Aldrich, USA). The oil phase is composed of light mineral oil (Sigma–Aldrich, USA) containing 2 wt.% Abil EM 90 (Evonik, Germany) that is used as a surfactant. The emulsion is produced by vortexing the aqueous and oil phases that have been mixed at a volume ratio of 1:6. The resulting emulsion drops are converted into microgels by exposing them to ultraviolet (UV) light at 100% intensity for 5 min (Omnicure S 1000, Lumen Dynamics, Canada) to initiate the free radical polymerization within drops. During the UV exposure, the emulsion was continuously stirred. The resulting microgel suspension was centrifuged at 4500 rpm (Mega Star 1.6R, VWR) for 10 min to remove the supernatant. This procedure was repeated four times with the microgels redispersed in ethanol (Sigma–Aldrich, USA) to remove the remaining oil. The washed microgels were vacuum‐dried at room temperature for a week.

### Synthesis of PATC Microgels

PATC microgels are prepared from water‐in‐oil emulsion templates. An aqueous phase containing 30 wt.% (3‐Acrylamidopropyl)trimethylammonium chloride (ATC) (Sigma–Aldrich, USA) and 4.5 mol% of MBAA and mineral oil containing 2 wt.% Abil EM 90 are mixed at a volume ratio of 1:6. This mixture is tip‐sonicated (Branson Ultrasonic, USA) with 30% energy for 1 min. To the resulting emulsion, 5 µL mL^−1^ of Irgacure 1173 was added that served as a photoinitiator. The emulsion was exposed to UV light for 5 min to initiate the free radical polymerization within the drops. Thereby, drops were converted into microgels. The microgels was washed as described above.

### Swelling Ratios of Microgels

The swelling ratios of the microgels are measured using confocal microscopy (Nikon Eclipse Ti2‐E inverted microscope). A microgel paste was prepared by soaking 0.05 g microgels in 1 mL of an aqueous solution containing 40 wt.% acrylamide (AAm) (Sigma–Aldrich, USA) and different concentrations of Sodium Chloride (NaCl) (Sigma–Aldrich, USA). To fluorescently label the microgels, 10 µg of fluorescein sodium salt (Sigma–Aldrich, USA) per mg of PATC microgels or 5 µg of Rhodamine B (Sigma–Aldrich, USA) per mg of PAA microgels was added to the AAm solution. The resulting paste was deposited between a glass slide and a cover glass separated by a 120 µm spacer. Three 80 µm z‐stacks of the samples were recorded with a step size of 0.63 µm, and the swelling ratio *r* is calculated by:

(4) $$r=\frac{V_{\mathrm{swollen}}}{V_{\mathrm{as}-\mathrm{prepared}}}\approx \frac{m_{\mathrm{swollen}}}{m_{\mathrm{as}-\mathrm{prepared}}}=\frac{\frac{0.63\times \sum A_{\mathrm{microgel}}}{80\times A}\times m_{\mathrm{paste}}\left(g\right)}{\frac{m_{\mathrm{microgel}\;}\left(g\right)}{W}}\;$$

where *A*_microgel_ is the thresholded area of the microgels seen on each slice, *A* is the imaging area, *m*_paste_ the mass of the microgel paste, *m*_microgel_ the mass of the dry microgel powder, and *W* the weight fraction of monomers in as‐prepared microgels, which is 0.3 in this case.

### Preparation of the Microgel Pastes

PAA and PATC microgels were mixed at the desired mass ratio. This mixture was lyophilized to obtain a microgel powder. The microgel powder was soaked overnight in an aqueous solution containing 40 wt.% Acrylamide (Sigma–Aldrich, USA), the desired concentration of MBAA that was used as a cross‐linker, 1 µL mL^−1^ Irgacure 1173, and the desired concentration of NaCl to obtain a microgel paste. The paste was centrifuged at 4500 rpm for 2 min to remove trapped air before further processing.

To ensure that the volume fraction of PAA and PATC microgels was close to 100%, thereby maximizing the contact area between the microgels, the precise amount of AAm solution was added to the microgel powder to obtain a microgel volume fraction of 100%. The volume of the AAm solution is calculated as follows:

(5) $$V_{\mathrm{AAm}}=V_{\mathrm{paste}}-V_{\mathrm{microgels}}\approx m_{\mathrm{paste}}-m_{\mathrm{microgels}}$$

(6) $$=\left(\frac{m_{PAA}\left(g\right)}{W_{\mathrm{PAA}}}\times r_{\mathrm{PAA}}+\frac{m_{\mathrm{PATC}}\left(g\right)}{W_{\mathrm{PATC}}}\times r_{\mathrm{PATC}}\right)-\left(m_{\mathrm{PAA}}\left(g\right)+m_{\mathrm{PATC}}\left(g\right)\right)$$

where *m*_PAA_ is the mass of the PAA microgel powder, *m*_PATC_ that of the PATC microgel powder, *W*_PAA_ and *W*_PATC_ are the initial weight fractions of AA or ATC in as‐prepared microgels, which is 0.3 in this case, *r_PAA_* is the swelling ratio of the PAA microgel powder in AAm solutions compared to the as‐prepared state, and *r_PATC_* is the swelling ratio of the PATC microgel powder in AAm solutions compared to the as‐prepared state.

### Fabrication of Molded DNGHs

To produce samples for simple extension tests, the microgel pastes were poured into Teflon molds with a dimension of 50 mm × 10 mm × 1 mm and covered by glass slides. For tensile tests, the paste was poured into dogbone‐shaped molds that had a gauge thickness of 1 mm, a width of 5 mm, and a gauge length of 10 mm. The microgel pastes were polymerized under UV light (CAMAG UV Lamp 4, 366 nm, 1 mW cm^−2^) for 10 min.

### Preparation of DN hydrogels

To prepare bulk double network hydrogels, an ATC/AA solution of the same composition as the microgel was photo‐polymerized to produce bulk PATC/PAA hydrogels with dimensions of 50 mm × 10 mm × 1 mm. The produced bulk hydrogels were swollen to equilibrium overnight in an aqueous solution containing 40 wt.% AAm, 0.2 mol% MBAA, 1 µL mL^−1^ Irgacure 1173, and 0, 0.1, 0.3, 0.5, 1 m NaCl. After removing the excess solution, the swollen hydrogels were illuminated with UV light for 10 min to initiate the free radical polymerization, resulting in bulk DN hydrogels.

### 3D Printing of DNGHs

The microgel pastes were loaded in a 3 mL Luer lock syringe and centrifuged at 4000 rpm for 1 min to remove air bubbles. The loaded ink was printed with a commercial 3D bioprinter (Inkredible+, Cellink). The pastes were extruded through a conical nozzle with a gauge size of 410 µm under a pneumatic pressure of 80 kPa. The printing paths were controlled by G‐codes generated using Cellink HeartWare software, with a writing speed set to 12 mm s^−1^. 3D structures were printed on a glass slide with a starting gap of 0.2 mm. The obtained structures were polymerized with UV light (CAMAG UV Lamp 4, 366 nm, 1 mW cm^−2^) for 10 min.

To evaluate the printing resolution of inks, lattices were printed with a dimension of 10 mm × 10 mm and quantify the shape fidelity *F* as:^[^ ^60^ ^]^

(7) $$F\left(\%\right)=\frac{A_{a}}{A_{t}}\times 100$$

where *A*_t_ is the theoretical area of a cell and *A*_a_ is the actual area of the cell.

To assess the shape stability of inks after polymerization, shape stability *St* is defined as:

(8) $$St\;\left(\%\right)=\frac{A_{a,c}-A_{a}}{A_{a}}\;\times 100$$

where *A*_**a**,**c**_ represents the area of the printed cell after polymerization, and *A*_**a**_ is the area before polymerization.

### Rheology of Inks

Rheological measurement was conducted using a DHR‐3 rheometer (TA Instruments) equipped with an 8 mm diameter parallel‐plate steel geometry. All experiments were carried out at 25 °C with a fixed gap of 1000 µm, and each measurement was repeated three times to ensure reproducibility. Frequency‐dependent viscosity measurements were performed at a strain amplitude of 0.5%, while shear stress sweeps were conducted at a fixed frequency of 1 Hz over a stress range of 1–1000 Pa.

### 3D Visualization of DNGHs

The 3D structure of DNGHs was visualized with confocal microscopy. Sheet‐like samples with a thickness of 120 µm were prepared by polymerizing a microgel paste sandwiched between two glass slides that had been separated with 120 µm spacers. To render the microgels fluorescent, the samples were stained by incubating them in an aqueous solution containing 1 µg mL^−1^ Fluorescein sodium salt (Sigma–Aldrich, USA) followed by an incubation in an aqueous solution containing 1 µg mL^−1^ Rhodamine B (Rho B) (Sigma–Aldrich, USA). The scanning area is 363.99 µm × 363.99 µm and the step size in the z‐direction is 0.63 µm. Z‐stacks with a thickness of 80 µm were recorded and reconstructed with ImageJ.

### Surface Roughness

Enamel roughness was measured to visualize the crack morphology in the fractured interfacially reinforced DNGH. Three specimens each were used for surface roughness measurement. The fractured DNGH sample was placed under the TS‐150 non‐contact profilometer and the surface was analyzed by Proscan software (Schaefer‐Tec AG, Switzerland). The scan set‐up parameters are as follows: scan rate = 300 Hz, average = 4 and step size = 0.004 mm.

### Uniaxial Tensile Test

Uniaxial tensile tests were performed on dogbone samples using a Universal Tensile Test Machine Zwicky 5 kN (Zwick Roell, Germany) with a velocity of 100 mm min^−1^. Young's moduli were calculated from a linear fit to the stress–strain curves between 2 and 10% strain. At least three samples are tested per experiment and the averages and standard deviations of the values are reported.

### Simple Extension Test (SET)

SET tests were performed on a Universal Tensile Test Machine Zwicky 5 kN (Zwick Roell, Germany) with a 50 N load cell. The sample measures 50 mm in length, 10 mm in height, and 1 mm in thickness. A 20 mm crack was at the midpoint of the short edge of the sample using a razor blade. The two arms of the samples were clamped with a hydraulic pressure of 2 bar and the samples were tested under mode Ι fracture with a velocity of 100 mm min^−1^. The reported fracture energy Γ is calculated as:^[^ ^25^ ^]^

(9) $$\Gamma =2\left(\frac{F\lambda }{t}-W\left(\lambda \right)H_{0}\right)$$

Here, *F* is the average of all force values on the plateau of the SET curve, λ the stretch of the legs at the plateau level of *F*, *t* the thickness of the sample, *W*(λ) the elastic strain energy at λ, and *H*_0_ the width of a leg. This formula accounts for the stretch in the legs during crack propagation, which becomes significant when the stretch falls within the range of 1.4 ≤ λ ≤ 2.2, as is the case for our samples. As the sample‐to‐sample variation of *h* and W(λ) within one data point are small compared to the variations within the first term of the equation, the second term of the equation is neglected and only consider the error from the first term. At least three samples are measured per data point.

### Dissipation Zone Size Measurement

The dissipation zone size of DNGHs is measured by the crack tip opening displacement (CTOD) method.^[^ ^46^ ^]^ Briefly, a crack of 7.5 mm was created at the edge of DNGH samples with a dimension of 15 mm × 20 mm × 1 mm using a razor blade. The cracked sample was placed under a Nikon optical microscope (Nikon AZ100, Japan). A far‐field mode I loading was applied to the sample using a self‐customized displacement‐controlled stage, with a loading speed of 100 mm min^−1^. Time‐lapse images of the sample were recorded with a fast‐speed camera (FASTCAM novas12, Japan) at 30 frames per second during the propagation of the crack. The images are thresholded with ImageJ, and the contour of the crack is extracted in a window of *x* × *y* = 6 × 12 mm. The crack tip is defined as the rightmost point on CTOD and the curve is analyzed using Python. At least three repeats are performed for each sample type and the averages and standard deviations are reported.

### Dissipation Zone Visualization

The dissipation zone around the crack tip was visualized using digital image correlation (DIC). The speckle pattern on the cracked DNGHs was created by spraying an ink (OBI Buntlack Spray, Switzerland) onto the hydrogel surface. The sample was subjected to a far‐field mode I loading with a loading speed of 100 mm min^−1^. The crack propagation was recorded with a fast camera at 30 frames per second. The obtained images are analyzed with Spam,^[^ ^61^ ^]^ with a subset radius of 10 pixels and a subset spacing of 10 pixels. At least three repeats are performed per sample type and the averages and standard deviations are reported.

### Contact Area and Volume Fraction Measurement

The contact area between PAA and PATC microgels and the microgel volume fractions in the samples are quantified from images acquired with a confocal microscope (Nikon Eclipse Ti2‐E inverted microscope, Japan). To fluorescently label the PAA microgels, 60 µg Rho B per mg of PAA microgels was added to the microgel paste. The resulting microgel paste was sandwiched between two glass slides separated by 120 µm spacers and illuminated with UV light (CAMAG UV Lamp 4, 366 nm, 1 mW cm^−2^) for 5 min to yield DNGH sheets. Z‐stacks of 80 µm thickness were recorded using a confocal microscope with a scanning area of 173.33 µm × 173.33 µm and a z‐direction step size of 0.63 µm. Each z‐slice is thresholded to estimate the contact area between oppositely charged microgels. This area is determined from the perimeter of the thresholded region and multiplied by the Z‐step size to calculate the volume of microgels per slice. The volume fractions are determined as the ratio between the volume of microgels and the scanned volume in the entire stack. For each dataset, three stacks are analyzed to obtain the average value.

### Peel Test

The adhesion energy between PAA and PATC DN hydrogels was measured via a 180° peel test performed on a Universal Tensile Test Machine Zwicky 5 kN (Zwick Roell, Germany) at a tensile rate of 100 mm min^−1^. The hydrogels were cut into rectangular samples with dimensions of 10 mm × 80 mm × 2 mm. The PATC DN hydrogel was attached to the surface of the PAA DN hydrogel by applying 5 N to the stacked DNs. The adhesion energy *E_a_* between hydrogels is calculated as $E_{a}=\frac{2F}{d}$, where *F* is the average of all force values on the plateau of the peel test curve and *d* is the width of the sample.

### Statistical Analysis

All experimental data are presented as mean values with error bars representing the standard deviation. Each measurement is conducted with n = 3 independent samples, and the reported values reflect the average across these replicates. Given the limited sample size, statistical hypothesis testing is not performed, as the results may not be statistically robust.

## Conflict of Interest

The authors declare no conflict of interest.

## Supporting information



Supporting Information

Supplemental Video 1

Supplemental Video 2

## Data Availability

The data that support the findings of this study are available from the corresponding author upon reasonable request.

## References

[1] B. R. Freedman , A. Kuttler , N. Beckmann , S. Nam , D. Kent , M. Schuleit , F. Ramazani , N. Accart , A. Rock , J. Li , M. Kurz , A. Fisch , T. Ullrich , M. W. Hast , Y. Tinguely , E. Weber , D. J. Mooney , Nat. Biomed. Eng 2022, 6, 1167. 34980903 10.1038/s41551-021-00810-0 PMC9250555

[2] H. Hong , Y. B. Seo , D. Y. Kim , J. S. Lee , Y. J. Lee , H. Lee , O. Ajiteru , M. T. Sultan , O. J. Lee , S. H. Kim , C. H. Park , Biomaterials 2020, 232, 119679. 31865191 10.1016/j.biomaterials.2019.119679

[3] X. Zhao , Soft Matter 2014, 10, 672. 24834901 10.1039/C3SM52272E PMC4040255

[4] X. Li , J. P. Gong , Nat. Rev. Mater. 2024, 9, 380.

[5] X. Zhao , X. Chen , H. Yuk , S. Lin , X. Liu , G. Parada , Chem. Rev. 2021, 121, 4309. 33844906 10.1021/acs.chemrev.0c01088 PMC9217625

[6] J. P. Gong , Soft Matter 2010, 6, 2583.

[7] Y. Zheng , J. Jiang , M. Jin , D. Miura , F. X. Lu , K. Kubota , T. Nakajima , S. Maeda , H. Ito , J. P. Gong , J. Am. Chem. Soc. 2023, 145, 7376. 36952244 10.1021/jacs.2c13764

[8] S. Ahmed , T. Nakajima , T. Kurokawa , Md. A. Haque , J. P. Gong , Polymer 2014, 55, 914.

[9] A. C. Daly , M. D. Davidson , J. A. Burdick , Nat. Commun. 2021, 12, 753. 33531489 10.1038/s41467-021-21029-2 PMC7854667

[10] S. C. Lee , G. Gillispie , P. Prim , S. J. Lee , Chem. Rev. 2020, 120, 10834. 32815369 10.1021/acs.chemrev.0c00015 PMC7673205

[11] M. Tang , Z. Zhong , C. Ke , Chem. Soc. Rev. 2023, 52, 1614. 36779285 10.1039/d2cs01011a

[12] S. Xin , K. A. Deo , J. Dai , N. K. R. Pandian , D. Chimene , R. M. Moebius , A. Jain , A. Han , A. K. Gaharwar , D. L. Alge , Sci. Adv. 2021, 7, eabk3087. 34652944 10.1126/sciadv.abk3087 PMC8519565

[13] M. Hirsch , A. Charlet , E. Amstad , Adv. Funct. Mater. 2021, 31, 2005929.

[14] D. B. Emiroglu , A. Bekcic , D. Dranseike , X. Zhang , T. Zambelli , A. J. deMello , M. W. Tibbitt , Sci. Adv. 2022, 8, eadd8570. 36525484 10.1126/sciadv.add8570 PMC9757745

[15] C. B. Highley , K. H. Song , A. C. Daly , J. A. Burdick , Adv. Sci. 2019, 6, 1801076. 10.1002/advs.201801076 PMC6325587 30643716

[16] A. Harada , R. Kobayashi , Y. Takashima , A. Hashidzume , H. Yamaguchi , Nat. Chem. 2011, 3, 34. 21160514 10.1038/nchem.893

[17] Y. L. Han , Y. Yang , S. Liu , J. Wu , Y. Chen , T. J. Lu , F. Xu , Biofabrication 2013, 5, 3. 10.1088/1758-5082/5/3/035004 23715009

[18] M. Asadikorayem , F. Surman , P. Weber , D. Weber , M. Zenobi‐Wong , Adv. Healthcare Mater. 2023, 13, 2301831. 10.1002/adhm.202301831 37501337

[19] C. Y. Li , D. K. Wood , C. M. Hsu , S. N. Bhatia , Lab Chip 2011, 11, 2967. 21776518 10.1039/c1lc20318e PMC3399244

[20] Q. Feng , D. Li , Q. Li , X. Cao , H. Dong , Bioactive Mater. 2021, 9, 105. 10.1016/j.bioactmat.2021.07.020 PMC8586262 34820559

[21] A. S. Caldwell , G. T. Campbell , K. M. T. Shekiro , K. S. Anseth , Adv. Healthcare Mater. 2017, 6, 1700254. 10.1002/adhm.201700254 PMC5550331 28485127

[22] D. R. Griffin , W. M. Weaver , P. O. Scumpia , D. Di Carlo , T. Segura , Nat. Mater. 2015, 14, 737. 26030305 10.1038/nmat4294 PMC4615579

[23] M. Kessler , T. Yuan , J. M. Kolinski , E. Amstad , Macromol. Rapid Commun. 2023, 44, 2200864. 10.1002/marc.202200864 36809684

[24] I. Kolvin , J. M. Kolinski , J. P. Gong , J. Fineberg , Phys. Rev. Lett. 2018, 121, 135501. 30312088 10.1103/PhysRevLett.121.135501

[25] R. Long , C.‐Y. Hui , Soft Matter 2016, 12, 8069. 27714361 10.1039/c6sm01694d

[26] T. L. Sun , T. Kurokawa , S. Kuroda , A. B. Ihsan , T. Akasaki , K. Sato , Md. A. Haque , T. Nakajima , J. P. Gong , Nat. Mater. 2013, 12, 932. 23892784 10.1038/nmat3713

[27] U. G. K. Wegst , M. F. Ashby , Philos. Mag. 2004, 84, 2167.

[28] D. Taylor , N. O'Mara , E. Ryan , M. Takaza , C. Simms , J. Mech. Behav. Biomed. Mater. 2012, 6, 139. 22301183 10.1016/j.jmbbm.2011.09.018

[29] J. F. M. Manschot , A. Brakkee , J. Biomech. 1986, 19, 511. 3745223 10.1016/0021-9290(86)90124-7

[30] F. Bono , S. H. Strässle Zuniga , E. Amstad , Adv. Funct. Mater. 2024, 35, 2413368.

[31] D. Zhao , Y. Liu , B. Liu , Z. Chen , G. Nian , S. Qu , W. Yang , ACS Appl. Mater. Interfaces 2021, 13, 13714. 33720679 10.1021/acsami.1c01413

[32] Q. Ge , Z. Chen , J. Cheng , B. Zhang , Y.‐F. Zhang , H. Li , X. He , C. Yuan , J. Liu , S. Magdassi , S. Qu , Sci. Adv. 2021, 7, eaba4261. 33523958 10.1126/sciadv.aba4261 PMC7787492

[33] Y. Wu , Y. Zeng , Y. Chen , C. Li , R. Qiu , W. Liu , Adv. Funct. Mater. 2021, 31, 2107202.

[34] J. Liu , J. Garcia , L. M. Leahy , R. Song , D. Mullarkey , B. Fei , A. Dervan , I. V. Shvets , P. Stamenov , W. Wang , F. J. O'Brien , J. N. Coleman , V. Nicolosi , Adv. Funct. Mater. 2023, 33, 2214196.

[35] Y. Jia , Z. Zhou , H. Jiang , Z. Liu , J. Mech. Phys. Solids 2022, 169, 105090.

[36] M. Wang , E. Bouchbinder , J. Fineberg , Phys. Rev. Lett. 2024, 133, 156201. 39454176 10.1103/PhysRevLett.133.156201

[37] X. Wei , C. Li , C. McCarthy , J. M. Kolinski , Nat. Phys. 2024, 20, 1009. 38882522 10.1038/s41567-024-02435-x PMC11178495

[38] H. Yin , D. R. King , T. L. Sun , Y. Saruwatari , T. Nakajima , T. Kurokawa , J. P. Gong , ACS Appl. Mater. Interfaces 2020, 12, 50068. 33085900 10.1021/acsami.0c15269

[39] M. V. Chin‐Purcell , J. L. Lewis , J. Biomech. Eng. 1996, 118, 545. 8950659 10.1115/1.2796042

[40] G. Han , U. Chowdhury , M. Eriten , C. R. Henak , Sci. Rep. 2021, 11, 9527. 33947908 10.1038/s41598-021-88942-w PMC8096812

[41] C. Li , X. Wei , M. Wang , M. Adda‐Bedia , J. M. Kolinski , J. Mech. Phys. Solids 2023, 178, 105330.

[42] A. A. Griffith , The Royal Society 1921, 221, 163.

[43] G. R. Irwin , J. Appl. Mech. 1957, 24, 361.

[44] T. Matsuda , R. Kawakami , T. Nakajima , J. P. Gong , Macromolecules 2020, 53, 8787.

[45] T. Matsuda , R. Kawakami , T. Nakajima , Y. Hane , J. P. Gong , Macromolecules 2021, 54, 10331.

[46] Y. Zheng , T. Matsuda , T. Nakajima , W. Cui , Y. Zhang , C.‐Y. Hui , T. Kurokawa , J. P. Gong , Proc. Natl. Acad. Sci., U. S. A. 2021, 118, e2111880118. 34848539 10.1073/pnas.2111880118 PMC8670445

[47] J. Zhao , K. Mayumi , C. Creton , T. Narita , J. Rheol. 2017, 61, 1371.

[48] E. Ducrot , Y. Chen , M. Bulters , R. P. Sijbesma , C. Creton , Science 2014, 344, 186. 24723609 10.1126/science.1248494

[49] S. Mzabi , D. Berghezan , S. Roux , F. Hild , C. Creton , J. Polym. Sci. B Polym. Phys. 2011, 49, 1518.

[50] S. Van Der Walt , J. L. Schönberger , J. Nunez‐Iglesias , F. Boulogne , J. D. Warner , N. Yager , E. Gouillart , T. Yu , PeerJ 2014, 2, e453. 25024921 10.7717/peerj.453 PMC4081273

[51] S. Friedowitz , A. Salehi , R. G. Larson , J. Qin , J. Chem. Phys. 2018, 149, 163335. 30384694 10.1063/1.5034454

[52] J. A. Lewis , J. E. Smay , J. Stuecker , J. Cesarano , J. Am. Ceram. Soc. 2006, 89, 3599.

[53] J. Montoya , J. Medina , A. Molina , J. Gutiérrez , B. Rodríguez , R. Marín , Addit. Manuf. 2021, 39, 101891.

[54] H. Wang , Z. Ouyang , L. Hu , Y. Cheng , J. Zhu , L. Ma , Y. Zhang , Food Chem. 2022, 397, 133725. 35908462 10.1016/j.foodchem.2022.133725

[55] A. Ribeiro , M. M. Blokzijl , R. Levato , C. W. Visser , M. Castilho , W. E. Hennink , T. Vermonden , J. Malda , Biofabrication 2017, 10, 014102. 28976364 10.1088/1758-5090/aa90e2 PMC7116103

[56] R. Zhao , A. Thoma , E. Amstad , Appl. Mater. Today 2024, 37, 102155.

[57] M. Hirsch , L. D'Onofrio , Q. Guan , J. Hughes , E. Amstad , Chem. Eng. J. 2023, 473, 145433.

[58] O. Guetta , B. H. Varfman , D. Rittel , J. Mech. Phys. Solids 2021, 146, 104220.

[59] P. Calvert , Adv. Mater. 2009, 21, 743.

[60] S. Bom , R. Ribeiro , H. M. Ribeiro , C. Santos , J. Marto , Int. J. Pharm. 2022, 615, 121506. 35085727 10.1016/j.ijpharm.2022.121506

[61] O. Stamati , E. Andò , E. Roubin , R. Cailletaud , M. Wiebicke , G. Pinzon , C. Couture , R. Hurley , R. Caulk , D. Caillerie , T. Matsushima , P. Bésuelle , F. Bertoni , T. Arnaud , A. Laborin , R. Rorato , Y. Sun , A. Tengattini , O. Okubadejo , J.‐B. Colliat , M. Saadatfar , F. Garcia , C. Papazoglou , I. Vego , S. Brisard , J. Dijkstra , G. Birmpilis , JOSS 2020, 5, 2286.

