# Supplementary material for: Electrostatically Reinforced Double Network Granular Hydrogels
Source: Adv Sci (Weinh). 2025 Apr 3;12(20):2412566. doi: 10.1002/advs.202412566 (PMC12120716; doi:10.1002/advs.202412566)
Supplement: Supplementary file 1 — Supporting Information [file ADVS-12-2412566-s003.pdf]

ADVANCED SCIENCE
Open Access

Supporting Information

for *Adv. Sci.*, DOI 10.1002/advs.202412566

Electrostatically Reinforced Double Network Granular Hydrogels

*Tianyu Yuan, Chenzhuo Li, John M. Kolinski and Esther Amstad**

Supporting Information

Electrostatically reinforced double network granular hydrogels

*Tianyu Yuan, Chenzhuo Li, John Kolinski, Esther Amstad**

Corresponding Author:*

Esther Amstad − Soft Materials Laboratory, Institute of

Materials, École Polytechnique Fédérale de Lausanne (EPFL), Lausanne 1015, Switzerland; orcid.org/0000-

0002-9491-1010; Email: esther.amstad@epfl.ch

Authors:

Tianyu Yuan − Soft Materials Laboratory, Institute of Materials, École Polytechnique Fédérale de Lausanne (EPFL), Lausanne 1015, Switzerland

Chenzhuo Li − Engineering Mechanics of Soft Interfaces Laboratory, Institute of Mechanical Engineering, École Polytechnique Fédérale de Lausanne (EPFL), Lausanne 1015, Switzerland

John Kolinski − Engineering Mechanics of Soft Interfaces Laboratory, Institute of Mechanical Engineering, École Polytechnique Fédérale de Lausanne (EPFL), Lausanne 1015, Switzerland

Supplementary Videos

Movie S1. Peeling test of double network hydrogel slabs

Movie S2. 3D printing of a bridge using microgel ink

Supplementary Figures

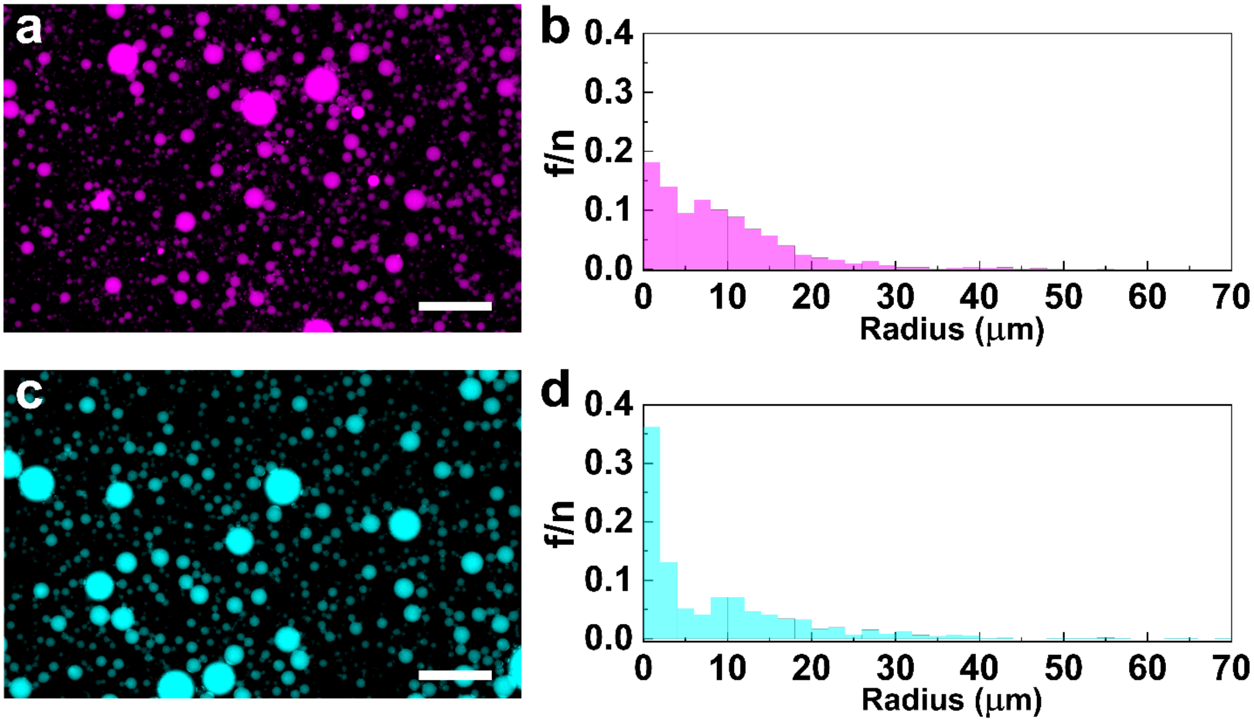

Figure S1. The fluorescence microscopy images of (a) PAA (magenta), and (c) PATC (cyan) microgels with (b, d) the corresponding size distributions. Scale bars are 300 μm.

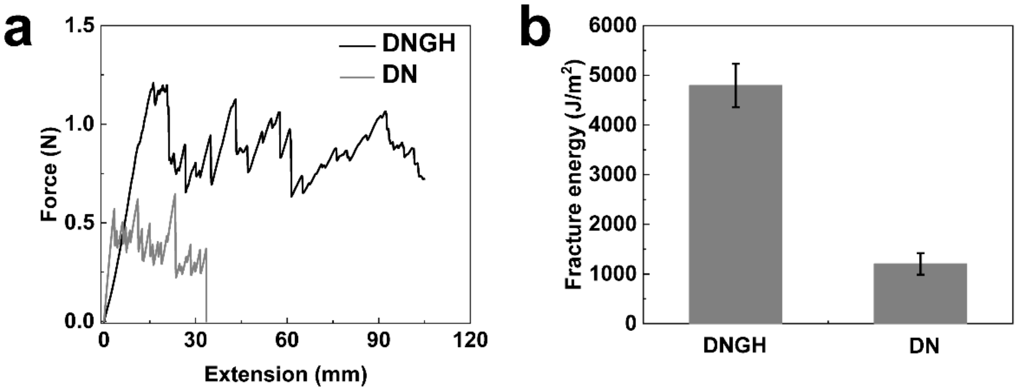

Figure S2. (a) Representative force-extension curves of the simple extension test (SET) performed on the interfacially reinforced DNGH containing 50 wt% PATC and 50 wt% PAA microgels and the double-network (DN) hydrogel with the same composition. (b) Fracture energy of DNGH and DN calculated from the plateau region on the curves in (a).

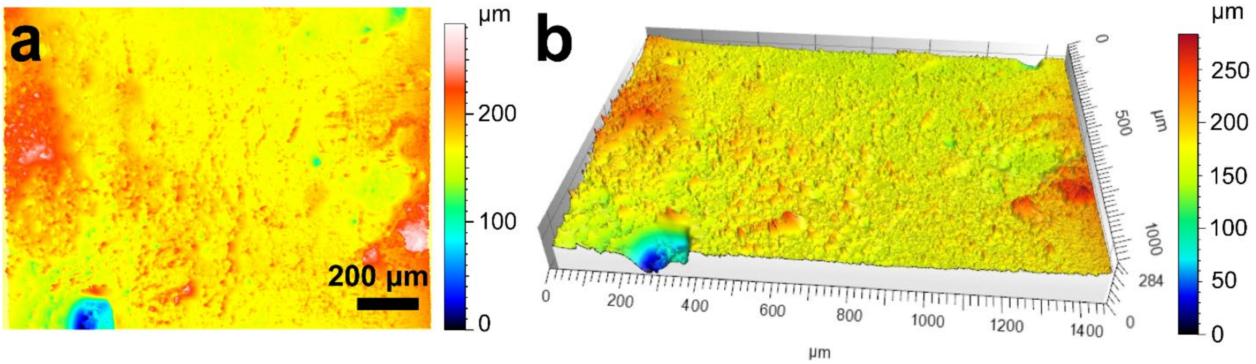

Figure S3. (a) 2D profile and (b) 3D reconstruction of the cut surface introduced by a razor blade in the DNGH.

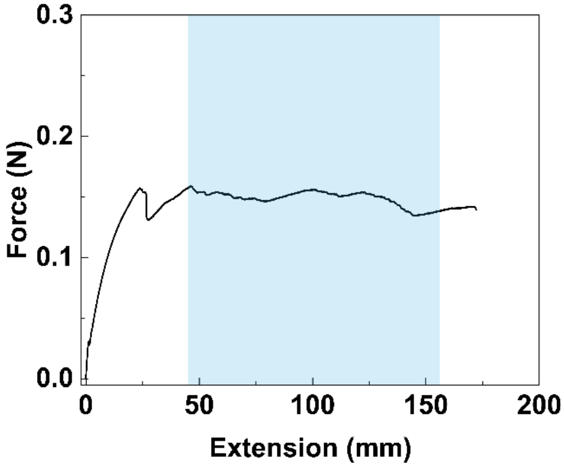

Figure S4. The representative force-extension curve of a simple extension test (SET) performed on the 2nd PAAm hydrogel containing 0.2 mol% MBAA. We calculate the fracture energy Γ_0 using the plateau regime of the curve, as indicated in the blue box. The calculated value is 566.0 ± 23.6 J/m^2.

Determine Γ_{inter} by SET

To verify whether electrostatic interactions influence the dissipative length scales, we quantify the elasto-adhesive length L_{EA} and the fracto-cohesive length L_{FC} of DNGHs containing only PATC microgels, only PAA microgels, or both microgels. These parameters are defined as:[1–5]

$$L_{EA} = \frac{\Gamma}{E} \quad (1)$$

$$L_{FC} = \frac{\Gamma}{W_f} \quad (2)$$

Where Γ is the fracture energy, E the elastic modulus, and W_f the work of fracture. All three groups exhibit similar L_{EA} and L_{FC} values, as summarized in **Table S1**. These results indicate that electrostatic interactions do not significantly alter the range of microgels that undergo damage. Therefore, we calculate Γ_{mg} from DNGH containing only PATC or PAA microgels and apply these values to DNGHs with both microgels.

To calculate the energy dissipated within microgels Γ_{mg}, we measure the fracture energy Γ_{PATC} or Γ_{PAA} of DNGHs containing only PATC or PAA microgels, as shown in **Figure S5**. The dissipated energy in PATC or PAA microgels $\Gamma_{mg,PATC}$ or $\Gamma_{mg,PAA}$ can be estimated as:

$$\Gamma_{mg,PATC} = \Gamma_{PATC} - \Gamma_0 \quad (3)$$

$$\Gamma_{mg,PAA} = \Gamma_{PAA} - \Gamma_0 \quad (4)$$

For DNGHs containing both types of microgels, Γ_{mg} is calculated using the rule of mixture and the calculated values of $\Gamma_{mg,PATC}$ and $\Gamma_{mg,PAA}$. The corresponding Γ_{inter} of DNGHs containing both types of microgels is given by:

$$\Gamma_{inter} = \Gamma - \Gamma_0 - \Gamma_{mg} \quad (5)$$

Where Γ is measured with SET as exemplified in **Figure S4**.

Table S1. Mechanical and fracture properties of DNGHs with different compositions.

	Γ (J/m^2)	W_f (MJ/m^3)	E (kPa)	L_{EA} (mm)	L_{FC} (mm)
$DNGH_{PATC}$	2904.4 ± 415.9	2.7 ± 0.3	221.7 ± 6.8	13.1 ± 1.9	1.1 ± 0.2
$DNGH_{PAA}$	1335.8 ± 187.4	1.6 ± 0.2	126.1 ± 7.5	10.6 ± 1.6	0.8 ± 0.2
$DNGH_{Mix}$	4791.4 ± 437.3	4.2 ± 0.6	333.2 ± 15.7	14.4 ± 1.5	1.1 ± 0.2

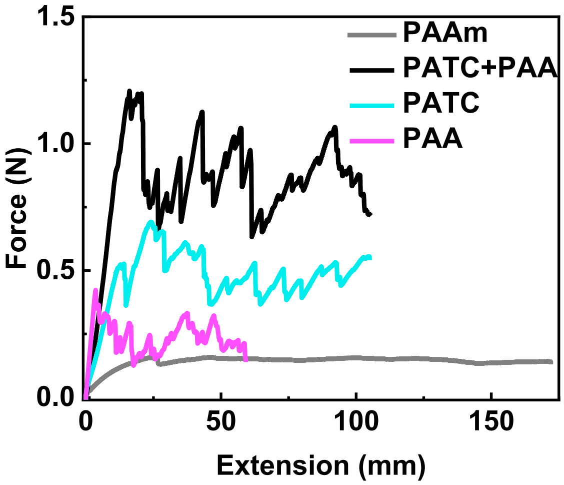

Figure S5. Representative force-extension curves of the simple extension test (SET) performed on the 2nd PAAm hydrogel containing 0.2 mol% MBAA, DNGHs containing 50 wt% PATC and 50 wt% PAA microgels, DNGHs containing only PATC microgels, and DNGHs containing only PAA microgels.

Derive the empirical equation for Γ_{inter}'

We expect Γ_{inter} to depend on the dissipated energy density U_{inter} within the dissipation zone with a radius of $\frac{L}{2}$. To test our hypothesis, we estimate Γ_{inter}' as:

$$\Gamma_{inter}' = \int_0^{\frac{L}{2}} U_{inter}(r)\, dr \tag{6}$$

To simplify the evaluation of Γ_{inter}', we assume U_{inter} remains constant within the dissipation zone and can be represented as the product of the adhesion energy E_a, which is the energy required per unit area to separate the oppositely charged microgels, and the surface-to-volume ratio $\frac{S}{V}$, where S represents the contact area between the oppositely charged microgels, and V is the volume of the region of interest.

$$U_{inter} = \frac{S}{V} \cdot E_a \tag{7}$$

Combining equations (1) and (2), we obtain the empirical equation $\Gamma_{inter}' = \frac{S}{V} \cdot E_a \cdot \frac{L}{2}$ introduced in the main text.

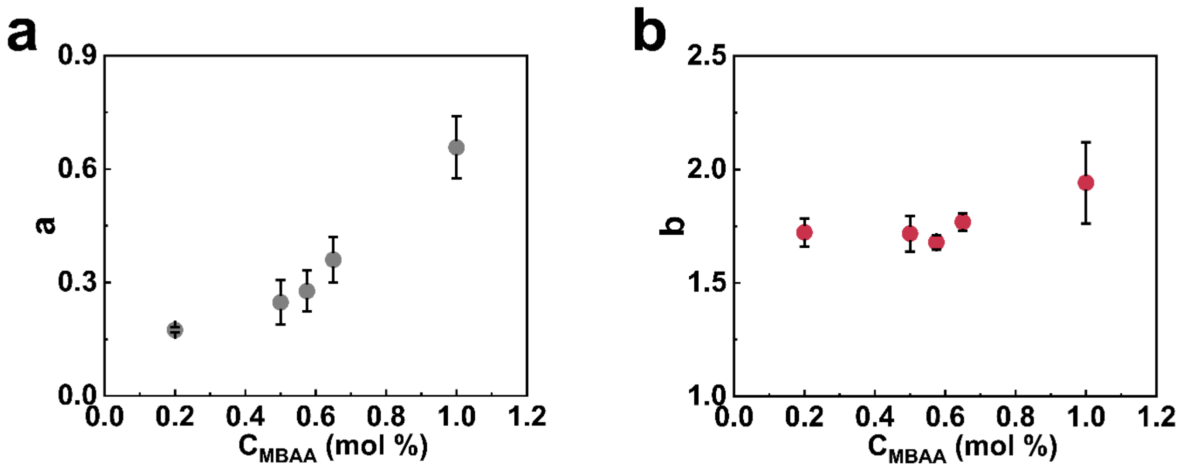

Figure S6. Quantification of the (a) a and (b) b values of interfacially reinforced DNGHs containing 0.2, 0.5, 0.575, 0.65, and 1 mol% MBAA in the 2nd network.

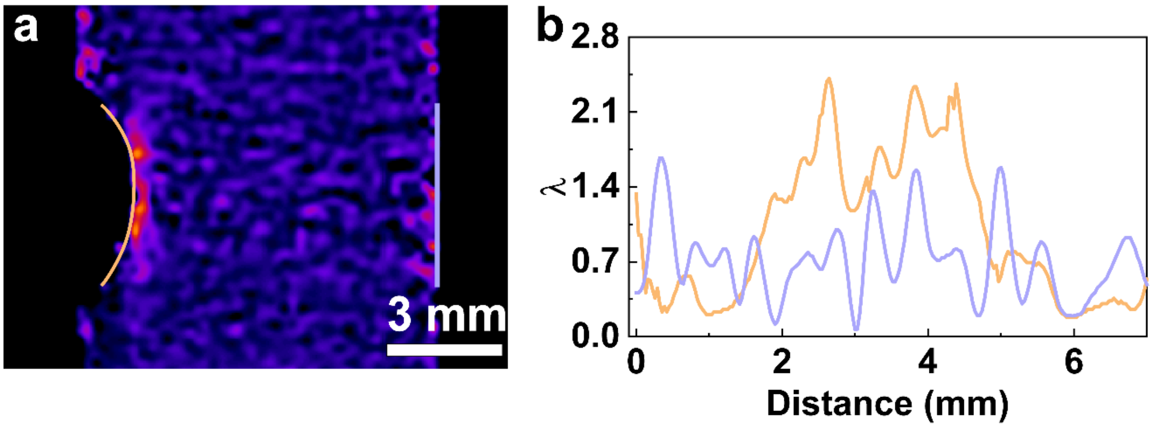

Figure S7. (a) Zoomed-in of the crack front and the sample edge in the digital image correlation (DIC) analysis. (b) Extracted principal stretch values around the crack tip (orange) and along the edge (purple) of the DNGH sample.

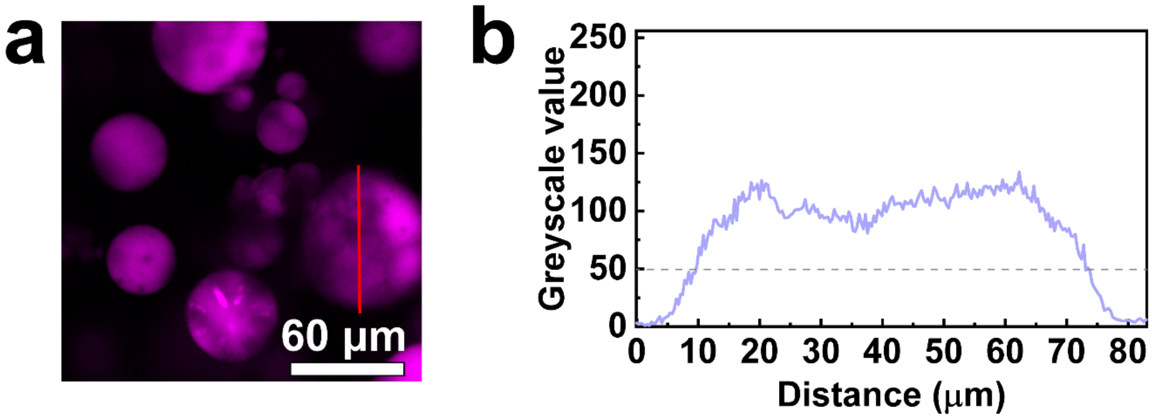

Figure S8. (a) A slice from the confocal Z-stack obtained for DNGH. (b) Greyscale intensity profile along the red line indicated in (a).

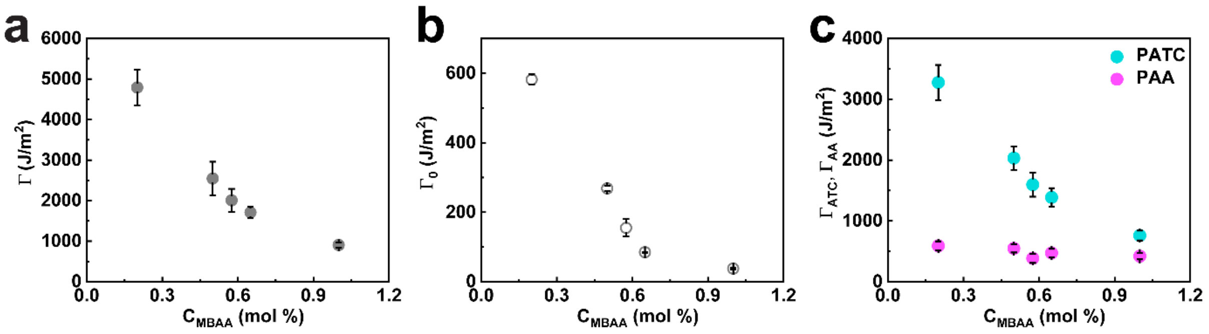

Figure S9. Fracture energy of (a) DNGHs containing 50 wt% PATC and 50 wt% PAA microgels, (b) secondary network, and (c) DNGHs containing only PATC or PAA microgels as a function of the MBAA concentration in the 2nd network.

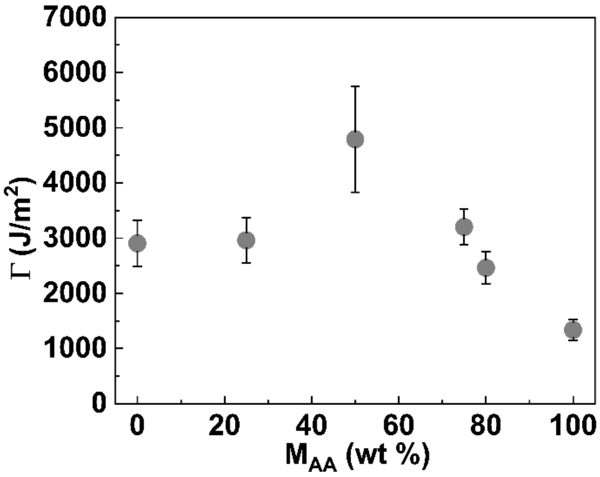

Figure S10. The fracture energy of DNGHs containing oppositely charged microgels as a function of the weight fraction of PAA microgels.

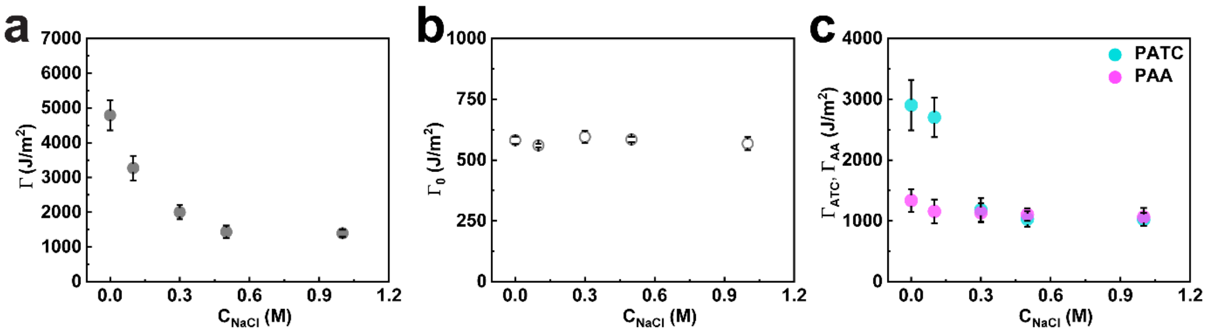

Figure S11. The fracture energy of (a) DNGHs containing 50 wt% PATC and 50 wt% PAA microgels, (b) secondary network, and (c) DNGHs containing only PATC or PAA microgels as a function of the NaCl concentration contained in them.

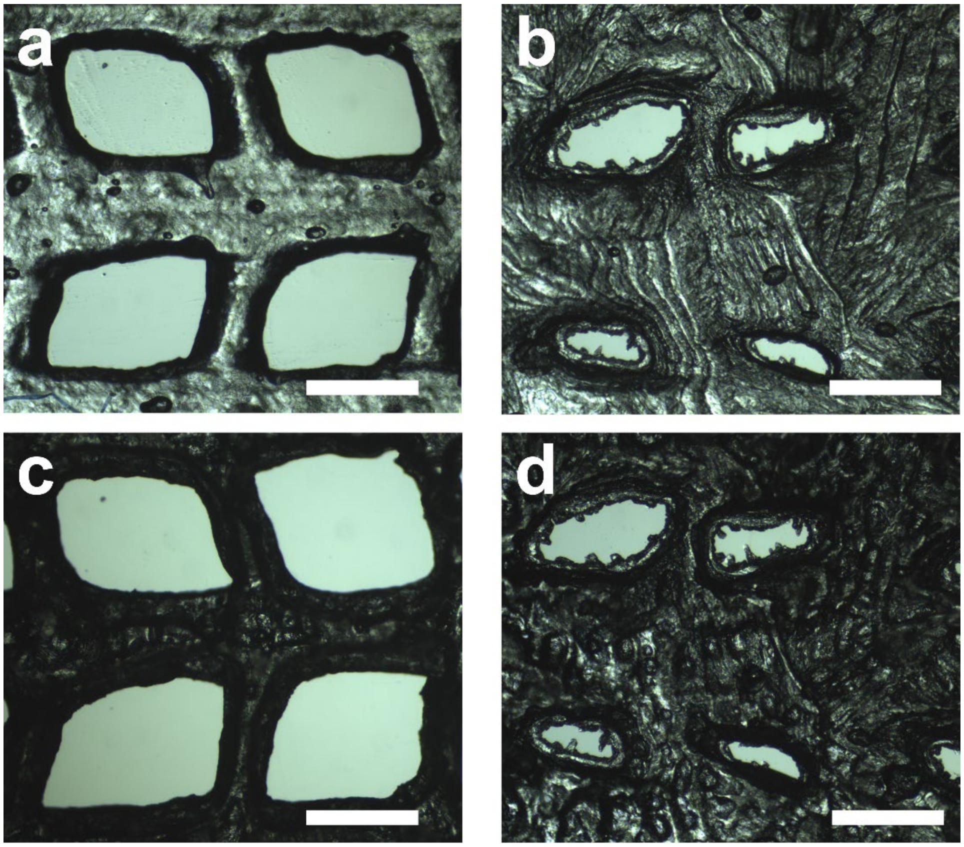

Figure S12. Optical microscope images of the printed lattice using inks containing (a) 0.1 M NaCl and (b) 1 M NaCl before curing, and (c) 0.1 M NaCl and (d) 1 M NaCl after curing. Scale bars represent 600 μm.

References

[1] C. Yang, T. Yin, Z. Suo, *Journal of the Mechanics and Physics of Solids* **2019**, *131*, 43.
[2] J. Liu, C. Yang, T. Yin, Z. Wang, S. Qu, Z. Suo, *Journal of the Mechanics and Physics of Solids* **2019**, *133*, 103737.
[3] C. Chen, Z. Wang, Z. Suo, *Extreme Mechanics Letters* **2017**, *10*, 50.
[4] R. Long, C.-Y. Hui, J. P. Gong, E. Bouchbinder, *Annu. Rev. Condens. Matter Phys.* **2021**, *12*, 71.
[5] C. Creton, M. Ciccotti, *Rep. Prog. Phys.* **2016**, *79*, 046601.
